# Genome wide association studies for body conformation traits in the Chinese Holstein cattle population

**DOI:** 10.1186/1471-2164-14-897

**Published:** 2013-12-17

**Authors:** Xiaoping Wu, Ming Fang, Lin Liu, Sheng Wang, Jianfeng Liu, Xiangdong Ding, Shengli Zhang, Qin Zhang, Yuan Zhang, Lv Qiao, Mogens Sandø Lund, Guosheng Su, Dongxiao Sun

**Affiliations:** 1 Key Laboratory of Animal Genetics and Breeding of Ministry of Agriculture, National Engineering Laboratory for Animal Breeding, College of Animal Science and Technology, China Agricultural University, 2 Yuanmingyuan West Road, Beijing 100193, China; 2 Beijing Dairy Cattle Center, Beijing 100192, China; 3 Life Science College, Heilongjiang Bayi Agricultural University, Daqing 163319, China; 4 Center for Quantitative Genetics and Genomics, Department of Molecular Biology and Genetics, Aarhus University, DK-8830 Tjele, Denmark

**Keywords:** Dairy cattle, GWAS, Body conformation traits, SNP, Holstein, QTL

## Abstract

**Background:**

Genome-wide association study (GWAS) is a powerful tool for revealing the genetic basis of quantitative traits. However, studies using GWAS for conformation traits of cattle is comparatively less. This study aims to use GWAS to find the candidates genes for body conformation traits.

**Results:**

The Illumina BovineSNP50 BeadChip was used to identify single nucleotide polymorphisms (SNPs) that are associated with body conformation traits. A least absolute shrinkage and selection operator (LASSO) was applied to detect multiple SNPs simultaneously for 29 body conformation traits with 1,314 Chinese Holstein cattle and 52,166 SNPs. Totally, 59 genome-wide significant SNPs associated with 26 conformation traits were detected by genome-wide association analysis; five SNPs were within previously reported QTL regions (Animal Quantitative Trait Loci (QTL) database) and 11 were very close to the reported SNPs. Twenty-two SNPs were located within annotated gene regions, while the remainder were 0.6–826 kb away from known genes. Some of the genes had clear biological functions related to conformation traits. By combining information about the previously reported QTL regions and the biological functions of the genes, we identified *DARC*, *GAS1*, *MTPN, HTR2A*, *ZNF521*, *PDIA6*, and *TMEM130* as the most promising candidate genes for capacity and body depth, chest width, foot angle, angularity, rear leg side view, teat length, and animal size traits, respectively. We also found four SNPs that affected four pairs of traits, and the genetic correlation between each pair of traits ranged from 0.35 to 0.86, suggesting that these SNPs may have a pleiotropic effect on each pair of traits.

**Conclusions:**

A total of 59 significant SNPs associated with 26 conformation traits were identified in the Chinese Holstein population. Six promising candidate genes were suggested, and four SNPs showed genetic correlation for four pairs of traits.

## Background

Since the 1990s, body conformation traits have been used in dairy cattle breeding programs in many countries. Although these traits themselves are not of economic interest to breeders, they are closely related to many economic traits, such as the health, productivity, and lifetime of cattle. Vollema et al. [1] reported that some conformation traits such as body depth, rump angle, rump width, and udder depth were useful predictors of lifetime and longevity in Dutch dairy bull populations because of the genetic correlation between them. Lund et al. [2] showed that genetic correlations between health and type traits were generally moderate (-0.32 to 0.37) and that selection for improved udder conformation reduced the somatic cell count and the occurrence of clinical mastitis. Short and Lawlor [3] found that genetic correlations between linear type traits and first lactation yield ranged from 0.48 to 0.54. Pozveh et al. reported that body depth had genetic correlations with many other economic traits, such as the days from calving to first-insemination (0.79), calving interval (0.35), and gestation length (0.34). Stature was also genetically correlated with gestation length (0.49) [4]. Therefore, quantitative trait loci (QTLs) associated with body conformation traits are economically as important as other economic traits.

With the availability of a high-density chip with single nucleotide polymorphisms (SNPs) for bovine, genome-wide association study (GWAS) has become a useful tool for fine-scale QTL mapping. This approach has been widely applied to causative mutation detection in human [5,6], mouse [7] and cattle [8,9]. By using very large numbers of SNPs researcher can easily detect statistical associations between SNPs and phenotypes, and thus biologically meaningful candidate genes close to the significant SNPs are identified for further study. This procedure greatly narrows down the regions of the genome that contain the causative mutations. The associations can provide direct and necessary evidence for the function of a gene.

Recently, many GWASs have been focused on the economic traits in dairy cattle, including production traits [8,10-15], fertility traits [8,16-18], disease resistance [9,19,20], and somatic cell score [13], and many statistically significant SNPs and biologically meaningful genes have been reported. However, comparatively few studies about body conformation traits have been published [8,21]. Linkage analysis has been used by some researchers to detect QTLs associated with conformation traits [22-24]. Schrooten et al. [22] used microsatellite markers in a whole genome scan for QTLs affecting 18 conformation traits. Ashwell et al. [23] detected QTLs affecting 22 conformation traits, including body, udder, feet, legs, and dairy conformation, and found 41 chromosome-wise significant QTLs. Cole et al. [8] used a single-locus model to analyze 18 body conformation traits , which included six trait groups, body size, body shape, udder, teats, teats, feet and legs, and final score and reported the top 100 effects for each trait. Their results showed that traits within a phenotype group had a tendency of sharing common SNP effects.

In this research, we performed a genome wide association study for 29 conformation traits in a Chinese Holstein population, which included 1314 Chinese Holstein cattle and 52,166 SNPs. A LASSO-like multiple-SNP method was applied to identify multiple SNPs simultaneously. The genes closest to the significant SNPs (within a 1 Mb region) were annotated.

## Methods

Blood samples were collected from Chinese Holstein cattle when the regular quarantine inspection of the farms was conducted. The procedure for collecting the blood samples was carried out in strict accordance with the protocol approved by the Animal Welfare Committee of China Agricultural University (Permit Number: DK996).

### Phenotype and genotype data

The Chinese Holstein population in this study comprised 1314 Chinese Holstein cows, the daughters of 22 sires. All the cows were from 22 dairy cattle farms in the Beijing Dairy Cattle Center and the Beijing Sanyuan Lvhe Dairy Farming Center where regular and standard performance tests, including measurement of conformation traits, have been carried out since 1999 as part of the Dairy Herd Improvement (DHI) system. According to the linear classification system defined by Dairy Data Center of China, Dairy Association of China (DAC) [25], 21 linear type traits were scored from 1 to 9, and eight composite traits were measured using an index with values and scored from 0 to 100. The 21 traits were animal size, stature, height at front end, chest width, body depth, loin strength, rump width, rump angle, bone quality, foot angle, rear legs side view, udder depth, udder texture, median suspensory, fore udder attachment, front teat placement, teat length, rear attachment height, rear attachment width, rear teat placement, and angularity. The eight function score traits were conformation (final score), dairy character, capacity, rump, feet and legs, fore udder, rear udder, and mammary system. Calculation of the scores for the eight composite traits was based on linear score, weights, and defective traits. The phenotypic values of the 21 conformation traits in the first lactation of the cows were measured by the Beijing Dairy Cattle Center [26] and then the genetic parameters of all 29 traits were estimated [27]. The estimated breeding values (EBVs) were calculated with a multiple-trait random regression test-day model using the RunGE software purchased from Canadian Dairy Network [28] by the Dairy Data Center of China. The descriptive statistics of the EBVs for the 29 traits in the 1314 individuals are listed in Table 1. The genetic correlations between each pair of traits were also calculated (see Additional file 1 for details). To conveniently generalize the results, the correlation coefficients (a total of 29*28/2 = 406 pair-wise values) were classified into five levels, -1 to -0.66, -0.66 to -0.33, -0.33 to 0.33, 0.33 to 0.66, and 0.66 to 1, and were defined as high-level negative correlation, medium-level negative correlation, weak-level correlation, medium-level positive correlation, high-level positive correlation, respectively (Table 2). The results show that 15 of 406 pairs of traits (7.2%) have high-level positive correlations, while most of the pairs have weak correlations (75.2%).

**Table 1:** Statistics of the estimated breeding values for the 29 conformation traits used in the GWAS

**Traits**	**N**	**Mean**	**Variance**	**SD**	**Min**	**Max**	**Heritability**
Conformation (final score)	1314	-1.58	9.73	3.12	-14	8	0.21
Capacity	1314	-1.92	14.61	3.82	-14	10	0.29
Stature	1314	-1.74	21.88	4.68	-25	13	0.37
Height at front end	1314	-0.96	8.42	2.90	-11	10	0.14
Animal size	1314	-1.50	15.27	3.91	-17	12	0.37
Chest width	1314	-2.59	14.30	3.78	-14	7	0.09
Body depth	1314	-0.84	13.76	3.71	-15	8	0.19
Rump	1314	-1.24	11.68	3.42	-12	11	0.07
Rump angle	1314	-0.52	18.87	4.34	-16	14	0.26
Rump width	1314	-0.87	24.09	4.91	-18	21	0.07
Loin strength	1314	-1.41	17.42	4.17	-19	11	0.17
Feet and legs	1314	-1.48	7.17	2.68	-11	7	0.09
Foot angle	1314	-1.03	12.11	3.48	-16	11	0.13
Bone quality	1314	-0.11	13.05	3.61	-14	10	0.10
Rear legs side view	1314	0.01	14.63	3.82	-17	13	0.24
Mammary system	1314	-0.81	13.06	3.61	-16	10	0.19
Udder depth	1314	-1.60	9.59	3.10	-15	8	0.22
Udder texture	1314	-1.17	8.39	2.90	-12	7	0.08
Median suspensory	1314	-0.44	15.10	3.89	-13	13	0.17
Fore udder	1314	-0.59	15.19	3.90	-16	12	0.17
Fore attachment	1314	-0.20	19.12	4.37	-15	11	0.27
Fore teat placement	1314	-0.64	13.37	3.66	-13	12	0.10
Teat length	1314	0.22	12.60	3.55	-16	11	0.18
Rear udder	1314	-0.70	13.16	3.63	-16	11	0.21
Rear attachment height	1314	-0.30	8.95	2.99	-11	8	0.15
Rear attachment width	1314	-1.23	11.34	3.37	-13	10	0.19
Rear teat placement	1314	-1.63	9.97	3.16	-12	9	0.11
Dairy character	1314	-1.50	16.40	4.05	-19	11	0.34
Angularity	1314	-1.39	12.22	3.50	-18	10	0.18

Note: N, SD, Min and Max means observations, standard deviation, minimum and maximum, respectively.

**Table 2:** Summary of the frequencies of pair-wise genetic correlations among 29 conformation traits

**Range**	**(-1 to -0.66)**	**(-0.66 to -0.33)**	**(-0.33 to 0.33)**	**(0.33 to 0.66)**	**(0.66 to 1)**	**Total**
**Number**	0	3	306	82	15	406
**Frequency**	0	0.007	0.752	0.201	0.072	1

The animals were genotyped using the Illumina BovineSNP50 BeadChip (Illumina Inc., San Diego, CA, USA). Some individuals were genotyped using the Illumina 54 K chip version1 containing 54,001 SNPs, while others were genotyped using the 54 K chip version 2 containing 54,609 SNPs. Genotype imputation was conducted for all the genotyped individuals using the Beagle software, version 3.1.0 [29,30]. After imputation, there were 56,270 SNPs in the marker data. SNPs were excluded from the analysis if the minor allele frequency (MAF) was less than 1%, the call rate was less than 90%, or the genotype frequency deviated from Hardy-Weinberg Equilibrium (HWE) with a *P*-value lower than 10^-6^. After quality control, 1314 individuals with 52,166 SNPs remained for the GWAS. After editing, the average distance between adjacent SNPs on the genome was 59.59 kb, and the median distance was 49.00 kb. Finally, the association analysis was conducted between each trait and 52,166 SNPs on 29 autosomes and X chromosome in the bovine genome.

### Statistical analyses

Statistical tests of SNP effects were conducted using the expectation maximization algorithm based on an improved least absolute shrinkage and selection operator (LASSO) [31] method. This method simultaneously estimates multiple SNP effects and shrinks the effects of zero-effect SNPs towards zero, and thus avoids complex model selection (Fang et al. 2013, unpublished).

The GWAS was carried out in two steps. First, single trait mixed model analysis (SMMA) was applied to estimate the effect of each SNP. Then, the first 500 markers (why 500 markers were used is explained in the Discussion section) with the lowest *P*-values were selected for the multiple-SNP analysis.

The linear model that was used to estimate the effect of the *j*th SNP can be expressed as:

(1) $$y=1\mu +x_{j}\beta_{j}+\mathrm{Zg}+e$$

where **y** is the vector of EBVs, **1** is the vector with its elements of 1, **μ** is the population mean; **x**_**j**_ is the vector of the genotype of the *j*th SNP marker, which is assigned to -1, 0, and 1 for genotypes AA, AB and BB, respectively, and **β**_*j*_is the SNP effect; **g** is the vector of polygenic effects, and **Z** is the design matrix related to the polygenic effect; **e** is the vector of random residuals. It is assumed that $g\sim N\left(0,A\sigma_{g}^{2}\right)$ and $e\sim N\left(0,I\sigma_{e}^{2}\right)$, where **A** is the additive genetic relationship matrix based on pedigree, $\sigma_{g}^{2}$ is the variance of polygenic effect, **I** is an identity matrix, and $\sigma_{e}^{2}$ is the residual variance. When a single-SNP mixed model was applied, the computational time was extremely large because of the iterative calculation of variance components (Best Linear Unbiased Prediction). Therefore, we first approximately calculated the variance components without considering a QTL effect and then imposed the estimates of variance components on the mixed model equation, which does not need an iterative calculation and thus reduces computational time.

For the SMMA analysis, the significance of the SNP effect was tested using a t-test with null hypothesis of β= 0, and the Bonferroni correction was applied to control the false positives. So, the threshold for significant associations was –log_10_ (0.05/*N*), where *N* is the number of SNP loci tested in the analysis.

The model to estimated effects of the SNPs selected from the first step can be expressed as:

(2) y=1μ+Xβ+Zg+e

where **X** is the matrix of genotype covariables of the 500 SNPs, and **β** is the vector of SNP effects. An expectation-maximization algorithm was adopted to estimate the model parameters. The method assigned an improved LASSO prior $\pi \left(\beta_{j}\right)=\frac{\lambda_{j}}{2}e^{-\lambda_{j}\left|\beta_{j}\right|}$ to SNP effect β_*j*_[32], where the hyper-parameter $\lambda_{j}^{2}/2$ is assigned a conjugate gamma prior with gamma (*a*,*b*), where *a* and *b* are very small values, and both *a* and *b* are taken as 10^-6^. The prior of the residual polygenic effect follows the normal distribution $g\left|\sigma_{g}^{2}\right)\sim N\left(0,A\sigma_{g}^{2}\right)$, where $\sigma_{g}^{2}$ is the residual polygenic variance and **A** is the additive genetic relationship matrix. The expectation-maximization algorithm was applied to estimate SNP effects **β**_*j*_ by finding the maximum posterior mode, which treats the polygenic effect (*g*) as a missing variable (see Additional file 2 for details).

The threshold value for declaring the significance of the SNP was determined from the empirical distribution of the heritability of SNP *j* (the SNP with the largest heritability across the genome for each permutation), $h_{j}^{2}=\sigma_{j}^{2}/\left(\sum_{j=1}^{p}\sigma_{j}^{2}+\sigma_{g}^{2}+\sigma_{e}^{2}\right)$, derived from 1,000 permutations, where $\sigma_{j}^{2}=2p_{j}\left(1-p_{j}\right)\beta_{j}^{2}$ is the variance of the *j*th SNP, and *p*_*j*_ is the allele frequency of the SNP. Here, heritability was used to measure the strength of each SNP, which is fairer than using the SNP effect, because the allele frequency of each SNP is different.

### Identification of SNP locations and gene annotation

The locations of significant SNPs were reported based on the UMD3.1 assembly of bovine genome sequence (assembled by the Center for Bioinformatics and Computational Biology (CBCB) at University of Maryland). The genes that were closest to the significant SNPs (within 1 Mb) were determined by the National Animal Genome Research Program [33] and the National Center for Biotechnology Information [34]).

## Results

A total of 59 genome-wise significant SNPs associated with 26 out of the 29 conformation traits were found by our improved LASSO method. Twenty-two of the SNPs were located within 22 known genes regions. We identified the 26 conformation traits into six trait group, and investigated the significant SNPs associated with each of these traits as described below.

### Dairy character traits

Three and two SNPs were associated with dairy character and angularity respectively (Table 3). Among them, dairy character and angularity shared one common SNP, which was located 45 kb away from *SLC25A24* on *Bos taurus* chromosome 3 (BTA3). For dairy character, one SNP was located within *SCEL* on BTA12 and the other SNP was 14 kb away from *SPATA17*. For angularity, the other SNP was 261 kb away from *HTR2A*.

**Table 3:** Genome-wide significant SNPs for final conformation score and dairy character traits

**Trait**	**SNP name**	**Chr.**	**Position (bp)**	**Nearest gene**	**Distance (bp)**	**Heritability**	**Threshold**
Conformation (final score)	ARS-BFGL-NGS-109711	5	110149999	*ANKRD54*	within	0.00980	0.00942
Dairy character	ARS-BFGL-NGS-14022	3	35255950	*SLC25A24*	45,501	0.01490	0.00929
	BTB-01238380	12	53100776	*SCEL*	within	0.00936	0.00929
	ARS-BFGL-NGS-55380	16	21821449	*SPATA17*	14,623	0.00990	0.00929
Angularity	ARS-BFGL-NGS-14022	3	35255950	*SLC25A24*	45,501	0.01100	0.00969
	ARS-BFGL-NGS-113826	12	17150394	*HTR2A*	261,113	0.01320	0.00969

Note: Heritability and threshold were obtained using the LASSO method. Nearest gene are symbols of gene full name in the NCBI database (http://www.ncbi.nlm.nih.gov/).

### Capacity traits

For body depth, height at front end, and animal size, each trait was associated with one significant SNP; for stature and loin strength, each trait was associated with two SNPs; and for chest width and capacity, each trait was associated with five SNPs (Table 4). Among them, the SNP on BTA3 was 7 kb away from *DARC* and was associated with both body depth and capacity; and the SNP on BTA25 was 9 kb away from *TMEM130*, and was associated with both body depth and animal size. The SNPs at 39 Mb on BTA9, 115 Mb on BTA6, 35 Mb on BTA15, 53 Mb on BTA12, and 10 Mb on BTA 18 were associated with capacity, stature, loin strength, height at front end, and chest width, respectively, and all of them were located in regions of the chromosomes that contained known genes. The remaining SNPs were at distances of 3 kb to 19 kb from the nearest known genes.

**Table 4:** Genome-wide significant SNPs for capacity and the component traits

**Trait**	**SNP name**	**Chr.**	**Position (bp)**	**Nearest gene**	**Distance (bp)**	**Heritability**	**Threshold**
Capacity	Hapmap40339-BTA-117016	3	10640386	*DARC*	7,094	0.01100	0.00894
	ARS-BFGL-NGS-114456	7	30964539	*LOC789456*	97,615	0.01100	0.00894
	ARS-BFGL-NGS-44162	9	39626344	*LOC539486*	within	0.01160	0.00894
	ARS-BFGL-NGS-26589	18	4852600	*NUDT7*	137,863	0.01080	0.00894
	ARS-BFGL-NGS-115067	25	37927752	*TMEM130*	8,967	0.01280	0.00894
Stature	Hapmap60794-rs29022851	6	115008971	*CPEB2*	within	0.01110	0.00953
	BTA-72885-no-rs	29	19560064	*LOC782090*	81,135	0.01370	0.00953
Body depth	Hapmap40339-BTA-117016	3	10640386	*DARC*	7,094	0.00884	0.00872
Loin strength	ARS-BFGL-NGS-70552	15	35177124	*SERGEF*	within	0.01340	0.00895
	BTB-00938945	26	32943986	*GPAM*	19,414	0.00908	0.00895
Height at front end	BTB-01238380	12	53100776	*SCEL*	within	0.00875	0.00811
Animal size	ARS-BFGL-NGS-115067	25	37927752	*TMEM130*	8,967	0.01130	0.00953
Chest width	BTA-110160-no-rs	8	81389800	*GAS1*	121,119	0.01870	0.00969
	ARS-BFGL-NGS-115466	18	10002426	*CDH13*	within	0.01410	0.00969
	BTA-45515-no-rs	19	43170256	*PTRF*	8,091	0.01220	0.00969
	BTB-00922140	4	82550244	*POU6F2*	54,944	0.01070	0.00969
	ARS-BFGL-NGS-57462	25	8086468	*LOC538487*	131,274	0.01030	0.00969

Note: Heritability and threshold were obtained using the LASSO method. Nearest gene are symbols of gene full name in the NCBI database (http://www.ncbi.nlm.nih.gov/).

### Rump traits

Eleven significant SNPs on different chromosomes were associated with rump traits (Table 5). Two and three of these SNPs were associated with rump and rump angle, respectively, and all of them were located within regions of the chromosomes that contained known genes. The remaining significant SNPs were at distances of 48 kb to 826 kb from the nearest known genes.

**Table 5:** Genome-wide significant SNPs for rump and the component traits

**Trait**	**SNP name**	**Chr.**	**Position (bp)**	**Nearest gene**	**Distance (bp)**	**Heritability/-log**_**10**_**(*P*)**^**b**^	**Threshold**
Rump	BTB-01660659	1	145986598	*KRTAP10-12*	688	0.01280	0.00916
	ARS-BFGL-NGS-12856	4	8155616	*CDK14*	within	0.01100	0.00916
	BTB-00323505	7	82338362	*ODZ2*	within	0.00966	0.00916
Rump width	BTB-00168895	4	20788689	*LOC781728*	166,306	0.01260	0.00917
	Hapmap40061-BTA-28737	9	1775187	*LOC616304*	826,933	0.00924	0.00917
	BTB-02035532^a^	7	58436123	*LOC100138639*	348,605	6.07^b^	6.02^c^
	ARS-BFGL-NGS-14128^a^	10	36665562	*ACYP2*	within	7.21^b^	6.02^c^
	ARS-BFGL-NGS-86147^a^	10	49856100	*ACYP2*	44,441	6.89^b^	6.02^c^
	ARS-BFGL-NGS-53281^a^	15	66603229	*SLC1A2*	within	8.35^b^	6.02^c^
	BTB-00611649^a^	15	67429625	*LDLRAD3*	within	6.05^b^	6.02^c^
	ARS-BFGL-NGS-97658^a^	15	68069900	*C15H11orf74*	158,748	7.27^b^	6.02^c^
	BTA-30189-no-rs^a^	X	60101130	*MAGED2*	42,513	6.34^b^	6.02^c^
	ARS-BFGL-NGS-80859^a^	X	61237718	*NXF3*	338,723	7.01^b^	6.02^c^
Rump angle	BTA-94299-no-rs	5	93940507	*MGST1*	within	0.01500	0.00906
	Hapmap48553-BTA-10000	7	59019641	*LOC788619*	36,977	0.01610	0.00906
	BTB-01219012	7	65799159	*LOC100296765*	48,625	0.01020	0.00906
	ARS-BFGL-NGS-31810	11	105631144	*LOC536255*	within	0.00960	0.00906
	ARS-BFGL-NGS-54462	25	13405791	*MIR365*	61,471	0.01190	0.00906
	ARS-BFGL-NGS-102900	27	4720968	*AGPAT5*	within	0.01300	0.00906

Note: Heritability and threshold were obtained using the LASSO method; -log_10_(*P*) was calculated using SMMA. ^a^SNP detected by SMMA only; ^b^-log_10_(*P*) obtained from SMMA; ^c^threshold of SMMA. Nearest gene are symbols of gene full name in the NCBI database (http://www.ncbi.nlm.nih.gov/).

### Feet and legs traits

Twelve significant SNPs were detected for feet and legs traits (Table 6). Three of these SNPs, for feet and legs, foot angle, and rear leg side view, were located within *DHX35* on BTA13, *PLEKHB2* on BTA2, and *DOCK10* on BTA2, respectively. Two SNPs on BTA3 and BTA27 for feet and legs, two SNPs on BTA1 and BTA15 for bone quality, three SNPs on BTA3, BTA4, and BTA22 for foot angle, two SNPs on BTA14 and BTA 24 for rear leg side view were located at distances of 3 kb to 420 kb from the nearest known genes.

**Table 6:** Genome-wide significant SNPs for feet and legs and the component traits

**Trait**	**SNP name**	**Chr.**	**Position (bp)**	**Nearest gene**	**Distance (bp)**	**Heritability/-log**_**10**_**(*P*)**^**b**^	**Threshold**
Feet and legs	Hapmap48847-BTA-67772	3	48281407	*RWDD3*	116,751	0.01530	0.00948
	ARS-BFGL-NGS-76581	27	39783292	*OXSM*	78,430	0.01290	0.00948
	Hapmap53251-rs29027216	13	68437003	*DHX35*	within	0.01050	0.00948
	Hapmap49594-BTA-39447^a^	1	20165566	*LOC101905904*	within	6.54^b^	6.02^c^
Bone quality	BTA-87372-no-rs	1	30724028	*LOC100337296*	420,082	0.00967	0.00949
	BTA-117758-no-rs	15	72591774	*C8H9orf30*	112,905	0.00964	0.00949
Foot angle	ARS-BFGL-NGS-18261	2	1896078	*PLEKHB2*	within	0.01010	0.00929
	ARS-BFGL-NGS-73625	3	14218748	*NES*	3,146	0.01060	0.00929
	Hapmap48448-BTA-71823	4	100663967	*MTPN*	37,399	0.00943	0.00929
	ARS-BFGL-NGS-113718	22	2655659	*CMC1*	29,461	0.01120	0.00929
Rear leg side view	ARS-BFGL-NGS-97763	2	113852386	*DOCK10*	within	0.01020	0.00942
	Hapmap29973-BTA-129162	14	46264806	*PAG1*	71,476	0.00978	0.00942
	UA-IFASA-4800	24	31524371	*ZNF521*	151,162	0.01230	0.00942
	Hapmap52451-rs29021142^a^	1	138784934	*KCNH8*	106,181	6.25^b^	6.02^c^

Note: Heritability and threshold were obtained using the LASSO method; -log_10_(*P*) was calculated using SMMA. ^a^SNP detected by SMMA only; ^b^-log_10_(*P*) obtained from SMMA; ^c^threshold of SMMA. Nearest gene are symbols of gene full name in the NCBI database (http://www.ncbi.nlm.nih.gov/).

### Mammary system traits

A total of 17 significant SNPs were detected for mammary system traits (Table 7). Of these SNPs, one associated with rear udder was located within *LOC100337279* on BTA14; two associated with udder texture were within *LOC100295233* and *DRG1* on BTA3 and BTA7, respectively; two associated with median suspensory fell were within *LRP2* and *MACROD2* on BTA2 and BTA13, respectively; one associated with fore teat placement was located within *SLC39A11* on BTA19; and one associated with rear teat placement was located within *SH3RF3* on BTA11. The other 10 SNPs were located at distances of 960 bp to 448 kb from the nearest known genes.

**Table 7:** Genome-wide significant SNPs for mammary system traits

**Trait**	**SNP name**	**Chr.**	**Position (bp)**	**Nearest gene**	**Distance (bp)**	**Heritability/-log**_**10**_**(*P*)**^**b**^	**Threshold**
Rear udder	ARS-BFGL-NGS-111920	14	44029634	*LOC100337279*	within	0.01330	0.00891
	Hapmap50827-BTA-94026	24	2166631	*LOC100336384*	39,890	0.01130	0.00891
Udder texture	ARS-BFGL-NGS-104839	3	88712390	*LOC100295233*	within	0.00873	0.00872
	BTA-41935-no-rs	17	72284836	*DRG1*	within	0.01670	0.00872
	BTB-01236227	20	15824409	*HTR1A*	264,560	0.00941	0.00872
Median suspensory	BTB-00089278	2	26942975	*LRP2*	within	0.01080	0.00874
	BTB-01007411	4	37145925	*SEMA3E*	960	0.00995	0.00874
	ARS-BFGL-NGS-35982	5	5693439	*NAP1L1*	81,318	0.00941	0.00874
	ARS-BFGL-NGS-29118	13	8497369	*MACROD2*	within	0.01490	0.00874
	ARS-BFGL-NGS-52278^a^	12	89182471	*RAB20*	within	7.85^b^	6.02^c^
Fore attachment	ARS-BFGL-NGS-114960	29	36024434	*NTM*	448,744	0.01050	0.00965
Fore teat Placement	ARS-BFGL-NGS-113245	19	59068269	*SLC39A11*	within	0.01290	0.00892
Teat length	BTB-01255458	10	99270875	*PDIA6*	80,295	0.01030	0.00911
Rear attach height	ARS-BFGL-NGS-20052	2	107616903	*CDK5R2*	3,609	0.00988	0.00904
	Hapmap43038-BTA-76203	6	50316616	*LOC100298058*	12,846	0.00997	0.00904
Rear attach Width	BTB-01478363	20	17370437	*BAG1*	210,690	0.00938	0.00924
Rear teat Placement	ARS-BFGL-NGS-31730	11	44265651	*SH3RF3*	within	0.00927	0.00864
	BTB-01230622	15	62600934	*DCDC5*	61,622	0.01200	0.00864

Note: Heritability and threshold were obtained using the LASSO method; -log_10_(*P*) was calculated using SMMA. ^a^SNP detected by SMMA only; ^b^-log_10_(*P*) obtained from SMMA; ^c^threshold of SMMA. Nearest gene are symbols of gene full name in the NCBI database (http://www.ncbi.nlm.nih.gov/).

### Final conformation score

A SNPs on BTA5 (Table 3) was found to be associated with final conformation score, and this SNP was harbored within *ANKRD54*, which encodes an ankyrin repeat domain-containing protein.

The estimated heritability for 29 conformation traits obtained using improved LASSO was plotted and the figures are available in Additional file 3.

The results obtained using SMMA are also listed in Tables 6, 7 and 8. Only 11 significant SNPs were detected and eight of them were significantly associated with rump width. The other three SNPs were associated with rear legs side view, median suspensory, and feet and legs.

**Table 8:** **Genome-wide significant SNPs compared with the SNPs reported by Cole et al.**[8]

**Chr.**	**Position (bp)**^**a**^	**Trait**^**a**^	**Position (bp)**^**b**^	**Distance (bp)**	**Trait**^**b**^
12	53100776	Dairy character	52240216	860,560	Teat length, Rear leg side view
16	21821449	Dairy character	21741980	79,469	Somatic cell score
16	21821449	Dairy character	22179897	358,448	Rear teat placement
16	21821449	Dairy character	22272329	450,880	Somatic cell score, Rear teat placement
16	21821449	Dairy character	22406467	585,018	Somatic cell score
18	4852600	Capacity	5655435	802,835	Foot angle
5	110149999	Conformation (final score)	110886859	736,860	Fore udder attachment, Rear udder height, Udder depth
5	110149999	Conformation (final score)	110910712	760,713	Fore udder attachment, Udder depth
7	30964539	Capacity	31136178	171,639	Somatic cell score
7	30964539	Capacity	31217950	253,411	Somatic cell score
7	30964539	Capacity	31655835	691,296	Teat length

^a^Results from our study; ^b^results reported by Cole et al. [8] Distance, the distance on the corresponding chromosome between the positions of the two SNPs (ours and the corresponding SNP from Cole et al.).

When we compared our results with those of Cole et al. [8] and Bolormaa et al. [21], we found that none of our significant SNPs were the same as the SNPs reported by Cole et al. [8] or Bolormaa et al. [21]. However, some of our SNPs were close to the significant SNPs reported by Cole et al. [8] that were associated with different traits (Table 8).

## Discussion

In this study, we performed a GWAS for 29 conformation traits in a population of Chinese Holstein cows. A two-step strategy was applied to estimate SNP effect, and first we selected 500 SNPs using SMMA. We originally planned to select SNPs with *P*-values < 0.01 (-log_10_(*P*) > 2), and we found that about 500 SNPs met this condition for the 29 traits (the -log_10_(*P*) values at the 500th marker were sorted into descending order for the 29 traits and ranged from 2.089 to 2.421). Therefore, we decided to use the top 500 SNPs for the multiple QTL analysis. In other words, the selected 500 SNPs include nearly all the SNPs with *P*-values < 0.01.

We found five SNPs located within previously reported QTL regions that were associated with conformation-related traits. The SNP on BTA12 associated with angularity is 261 kb away from *HTR2A* and is located within a QTL region that has been reported by Schrooten et al. [22] to be associated with angularity. The SNP on BTA29 associated with stature is 81 kb away from *LOC782090* and is within a large QTL region that has been found to significantly affect Angus body height at maturity [35]. The SNP on BTA24 associated with rear leg side view is near *ZNF521* and is within a QTL region that has been reported to have a significant effect on dairy cattle rear leg set [22]. The SNP on BTA10 associated with teat length is near *PDIA6* and is located within a QTL region that has been shown to have a significant effect on teat length [36]. And, the SNP on BTA25 associated with animal size is near *TMEM130* and is within a QTL region that has been reported to affecting calf size in Danish Holstein cattle [37]. Besides, most of significant SNPs that we detected in this study are located within QTL regions that have been reported previously to affect production, longevity, and reproduction traits in dairy cattle [21,35,36,38,39].

We also found several SNPs located within genes that are known to have functions related to the development and metabolism of animal tissues. The SNP (Hapmap40339-BTA-117016; Table 4) on BTA3 which was associated with both capacity and body depth is 7 kb away from the gene, Duffy blood group, chemokine receptor *(DARC)*. Hai et al. [40] performed a bivariate GWAS in human to identify the SNPs associated with lean body mass and age at menarche and reported that *DARC* may play an important role in regulating the metabolisms of both these traits. The SNP (BTA-110160-no-rs; Table 4) on BTA8 associated with chest width is 121 kb away from the growth arrest specific 1 *(GAS1)* gene. *GAS1* is highly expressed in quiescent mammalian cells and its over-expression in normal and some cancer cell lines was reported to inhibit G0/G1 transition [41]. It was found that *GAS1* was expressed by chondrocytes after the cartilage started to differentiate [41]. The SNP on BTA4 associated with foot angle is 37 kb away from the myotrophin (*MTPN*) gene, which plays an important role in cell and skeletal muscle growth [42]. These genes are suggested as functional candidate genes for body conformation traits.

Generally, different SNPs are associated with different traits, but some SNPs have been found to affect more than one trait. In our study, SNP Hapmap40339-BTA-117016 (Table 4) was associated with both capacity and body depth, SNP ARS-BFGL-NGS-115067 (Table 4) was associated with both capacity and animal size, SNP ARS-BFGL-NGS-14022 (Table 3) was associated with both dairy character and angularity, and SNP BTB-01238380 (Tables 3 and 4) was associated with both dairy character and height at front end. The genetic correlation between each of these pairs of genes was 0.51, 0.77, 0.86, and 0.35, which suggested that these four SNPs likely contribute to genetic correlation and perhaps have a pleiotropic effect on each pair of traits.

## Conclusions

The present genome-wide association study identified 59 significant SNPs associated with 26 conformation traits in a Chinese Holstein cattle population. Some of these SNPs were located within or near previously reported genes and QTL regions, while some of the SNPs were new discoveries. We found that *DARC*, *GAS1*, *MTPN, HTR2A*, *ZNF521*, *PDIA6*, and *TMEM130* were the most promising candidate genes for capacity and body depth, chest width, foot angle, angularity, rear leg side view, teat length, and animal size traits, respectively.

## Abbreviations

GWAS: Genome-wide association study; EBV: Estimate breeding value; SNP: Single-nucleotide polymorphism; QTL: Quantitative trait locus; BTA: *Bos taurus* automosome; LASSO: Least absolute shrinkage and selection operator; DAC: Dairy Association of China; DHI: Dairy Herd Improvement system; MAF: Minor allele frequency; SMMA: Single trait mixed model analysis; GAS1: Growth arrest specific 1; HTR2A: 5-hydroxytryptamine (serotonin) receptor 2A; ANKRD54: Ankyrin repeat domain 54; DHX35: DEAH (Asp-Glu-Ala-His) box polypeptide 35; DOCK10: Dedicator of cytokinesis 10; DRG1: Developmentally regulated GTP binding protein 1; DARC: Duffy blood group, chemokine receptor; LRP2: Low density lipoprotein receptor-related protein 2; MACROD2: MACRO domain containing 2; MTPN: Myotrophin; PDIA6: Protein disulfide isomerase family A, member 6; SCEL: Sciellin; SH3RF3: SH3 domain containing ring finger 3; SLC25A24: Solute carrier family 25 (mitochondrial carrier; phosphate carrier): Member 24; SLC39A11: Solute carrier family 39 (metal ion transporter), member 11; SPATA17: Spermatogenesis associated 17; TMEM130: Transmembrane protein 130; ZNF521: Zinc finger protein 52.

## Competing interests

The authors declare that they have no competing interests.

## Authors’ contributions

XW performed the genome-wide association analysis and prepared the manuscript. MF contributed to the statistical and results analysis, and contributed to manuscript writing. SW contributed to genotype imputation on v1 and v2 50 K chips. JL and XD participated in the data analysis. SZ participated in the data analysis and experiment design, LL and LQ performed the conformation trait measurements and EBV estimation. QZ and YZ participated in the experiment design and result interpretation. LM and GS revised the manuscript and participated in interpreting the result. DS conceived and designed the experiments and prepared the manuscript. All authors read and approved the final manuscript.

## Supplementary Material

Additional file 1 **Pair-wise genetic correlation for 29 conformation traits in 1314 Chinese Holstein cattle.** This file contains a table that lists the genetic correlation for 29 conformation traits. Click here for file

Additional file 2 Expectation-maximization algorithm with improved LASSO prior. Click here for file

Additional file 3 **Heritability estimates of the SNPs in LASSO analysis for the 29 investigated conformation traits.** This file contains the figures of the heritability estimates of the SNPs in LASSO analysis for 29 investigated conformation traits with thresholds (dotted lines) ascertained from 1,000 permutations. Five hundred SNPs against the heritability of 29 traits are plotted. Click here for file
