# Supplementary material for: Genome wide association studies for body conformation traits in the Chinese Holstein cattle population
Source: BMC Genomics. 2013 Dec 17;14:897. doi: 10.1186/1471-2164-14-897 (PMC3879203; doi:10.1186/1471-2164-14-897)
Supplement: Additional file 2 — Expectation-maximization algorithm with improved LASSO prior. [file 1471-2164-14-897-S2.doc]

**EM algorithm under improved LASSO prior**

**Prior specifications**

The population mean follows a uniform prior, $\pi(\mu)\propto1$. We have modified the LASSO prior by assigning an independent double-exponential prior to each marker effect [1], *i.e.*,

$\pi(\text{β}_{j})=\frac{\lambda_{j}}{2}e^{-\lambda_{j}\left|\beta_{j}\right|}$, (1)

which can be factorized into two-level priors: at the first level, $\text{β}_{j}$follows a normal distribution,

$\text{β}_{j}\left|\text{τ}_{j}^{2}\right.\sim N(0,\text{τ}_{j}^{2})$; (2)

and at the second level, $\text{τ}_{j}^{2}$ follows an exponential distribution,

$\pi(\text{τ}_{j}^{2}\left|\lambda_{i}\right.)=\text{Expon}\left(\text{τ}_{j}^{2}\left|\frac{\lambda_{i}^{2}}{2}\right.\right)=\frac{\lambda_{i}^{2}}{2}e^{-\lambda_{i}^{2}\text{τ}_{j}^{2}/2}$, (3)

where $\lambda_{j}$ is the hyper parameter and $\lambda_{j}^{2}/2$ is assigned a conjugate Gamma prior, Gamma(*a*,*b*) with *a* and *b* being very small numbers, and here both *a* and *b* are taken as 10^-6^. The special prior was to have special characters in that it could estimate zero-effect marker effect very close to zero[1]. The prior of the residual variance $\sigma^{2}$ follows non-informative scale-invariant prior $\pi(\sigma_{e}^{2})\propto1/\sigma_{e}^{2}$; the prior of the residual polygenic effect follows normal distribution $g\left|\sigma_{g}^{2}\right.\sim N(0,A\sigma_{g}^{2})$, where $\sigma_{g}^{2}$ is the residual polygenic variance and *A* is the additive genetic relationship matrix, which can be inferred from pedigree.

**EM algorithm**

Let $\Lambda=\left\{\lambda_{j}^{2}\right\}_{j=1}^{p}$, $\Sigma=\left\{\tau_{j}^{2}\right\}_{j=1}^{p}$, and $\theta=\left(\sigma_{e}^{2},\mu,\Lambda,\Sigma\right)$; then the likelihood can be expressed as

$\begin{matrix} L(\theta\left|\beta,y\right.)=p(y\left|\beta,\sigma_{e}^{2},\mu\right.)p(\beta\left|\Sigma\right.)p(\left.\Sigma\right|\Lambda)p(\Lambda)p(g\left|\sigma_{g}^{2}\right.) \\ \propto(\sigma_{e}^{2})^{-n/2}\exp\left[-\frac{1}{2\sigma_{e}^{2}}(y-\mu-X\beta-g)'(y-\mu-X\beta-g)\right] \\ \cdot\prod_{j=1}^{p} \text{τ}_{j}^{-1}\exp\left[-\frac{\beta_{j}^{2}}{2\text{τ}_{j}^{2}}\right]\cdot\prod_{j=1}^{p} \frac{\lambda_{j}^{2}}{2}\exp\left[-\frac{\lambda_{j}^{2}}{2}\text{τ}_{j}^{2}\right] \\ \\ \cdot\prod_{j=1}^{p} b^{a}\left[\frac{\lambda_{j}^{2}}{2}\right]^{a-1}\exp\left[-b\frac{\lambda_{j}^{2}}{2}\right]\cdot(\sigma_{g}^{2})^{-n/2}\exp\left[-\frac{1}{2\sigma_{g}^{2}}g'A^{-1}g\right]. \\ \end{matrix}$ (4)

*E-step*

Since the polygenic effect *g* cannot be derived explicitly, it is treated as missing data here and substituted with its posterior expectation

$E\left(g\left|y,\theta\right.\right)=\left(Z'Z+A^{-1}\frac{\sigma_{e}^{2}}{\sigma_{g}^{2}}\right)^{-1}Z'\left(y-\mu-X\beta\right)$. (5)

The posterior variance of *g* is $\text{Var}\left(g\left|y,\theta\right.\right)=\left(Z'Z+A^{-1}\frac{\sigma_{e}^{2}}{\sigma_{g}^{2}}\right)^{-1}\sigma_{e}^{2}$. (6)

*M-step*

The M-step maximizes the logarithm of the likelihood (6) $\log\left(L(\theta\left|\beta,y\right.)\right)$ with respect to $\mu$, $\text{τ}_{j}^{2}$, $\lambda_{j}^{2}$, $\sigma_{e}^{2}$and $\sigma_{g}^{2}$ to obtain the their next estimates,

$\mu=1'_{n}(y-\sum_{j=1}^{p} X_{j}\beta_{j})/n$ (7)

$\text{τ}_{j}^{2}=\frac{\sqrt{1+4\lambda_{j}^{2}\beta_{j}^{2}}-1}{2\lambda_{j}^{2}}$, (8)

$\lambda_{j}^{2}=\frac{a}{\text{τ}_{j}^{2}+b}$, (9)

$\begin{matrix} \sigma_{e}^{2}=\frac{1}{n}\left(y-\mu-\sum_{j=1}^{p} X_{j}\beta_{j}\right)^{'}\left(y-\mu-\sum_{j=1}^{p} X_{j}\beta_{j}-g\right) \\ +\frac{1}{n}\text{tr}\left(A^{-1}V_{g}\right), \end{matrix}$ (10)

and $\sigma_{g}^{2}=\frac{1}{n}\left[g'A^{-1}g+\text{tr}(A^{-1})V_{g}\right]$, (11)

where $V_{g}=\text{Var}\left(g\left|y,\theta\right.\right)$.

Given the initial values for $\theta$, the EM algorithm proceeds with repeatedly updating the E-step equations (5) and (6) and the M-step equations (7)-(11) until reaching convergence.

**References**

1. Fang M, Jiang D, Li D, Yang R, Fu W, et al. (2012) Improved LASSO priors for shrinkage quantitative trait loci mapping. Theor Appl Genet 124: 1315-1324.
