# Supplementary material for: Genome wide association studies for body conformation traits in the Chinese Holstein cattle population
Source: BMC Genomics. 2013 Dec 17;14:897. doi: 10.1186/1471-2164-14-897 (PMC3879203; doi:10.1186/1471-2164-14-897)

## Slide 1
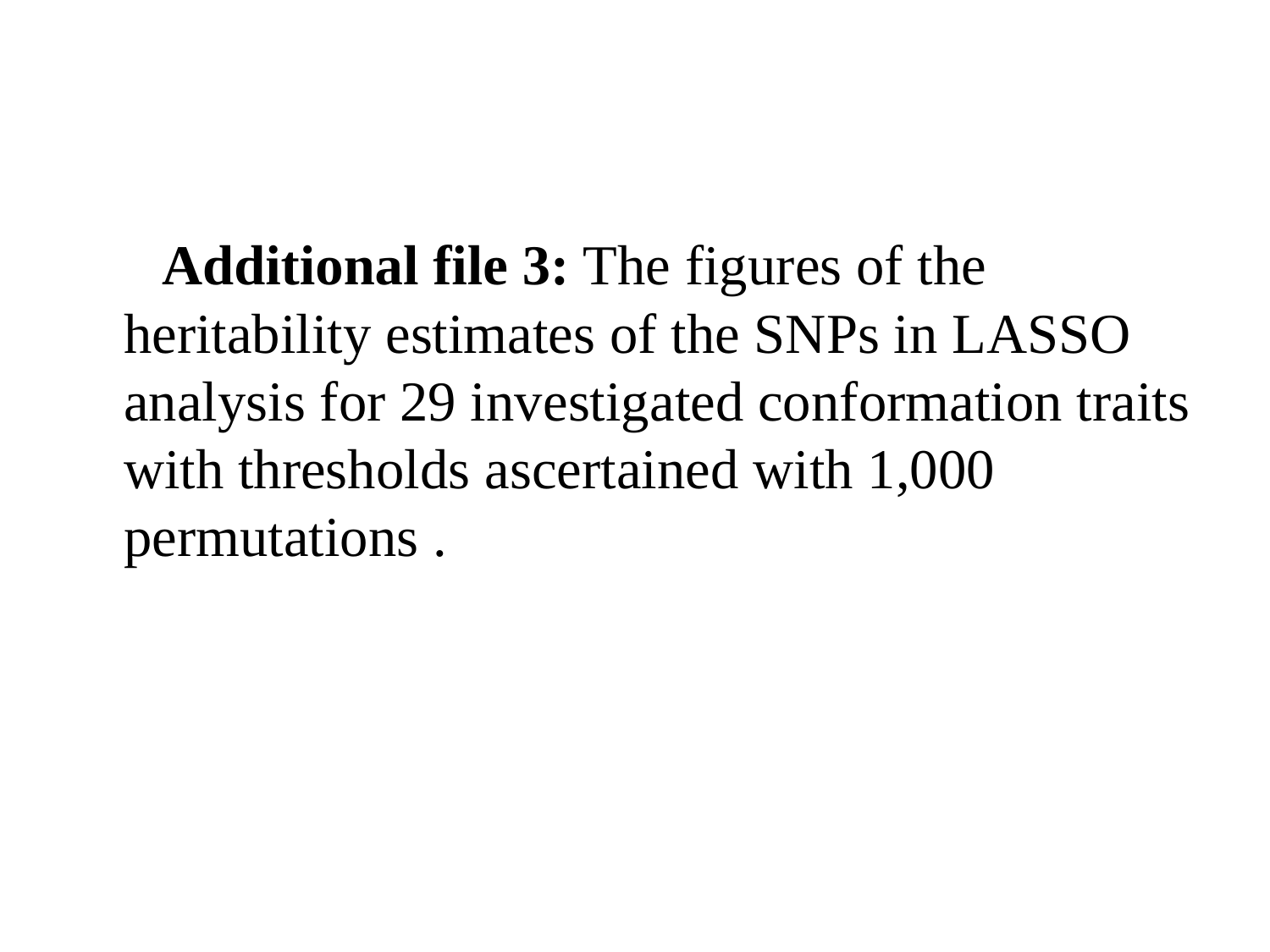

#
 Additional file 3: The figures of the heritability estimates of the SNPs in LASSO analysis for 29 investigated conformation traits with thresholds ascertained with 1,000 permutations .

## Slide 2
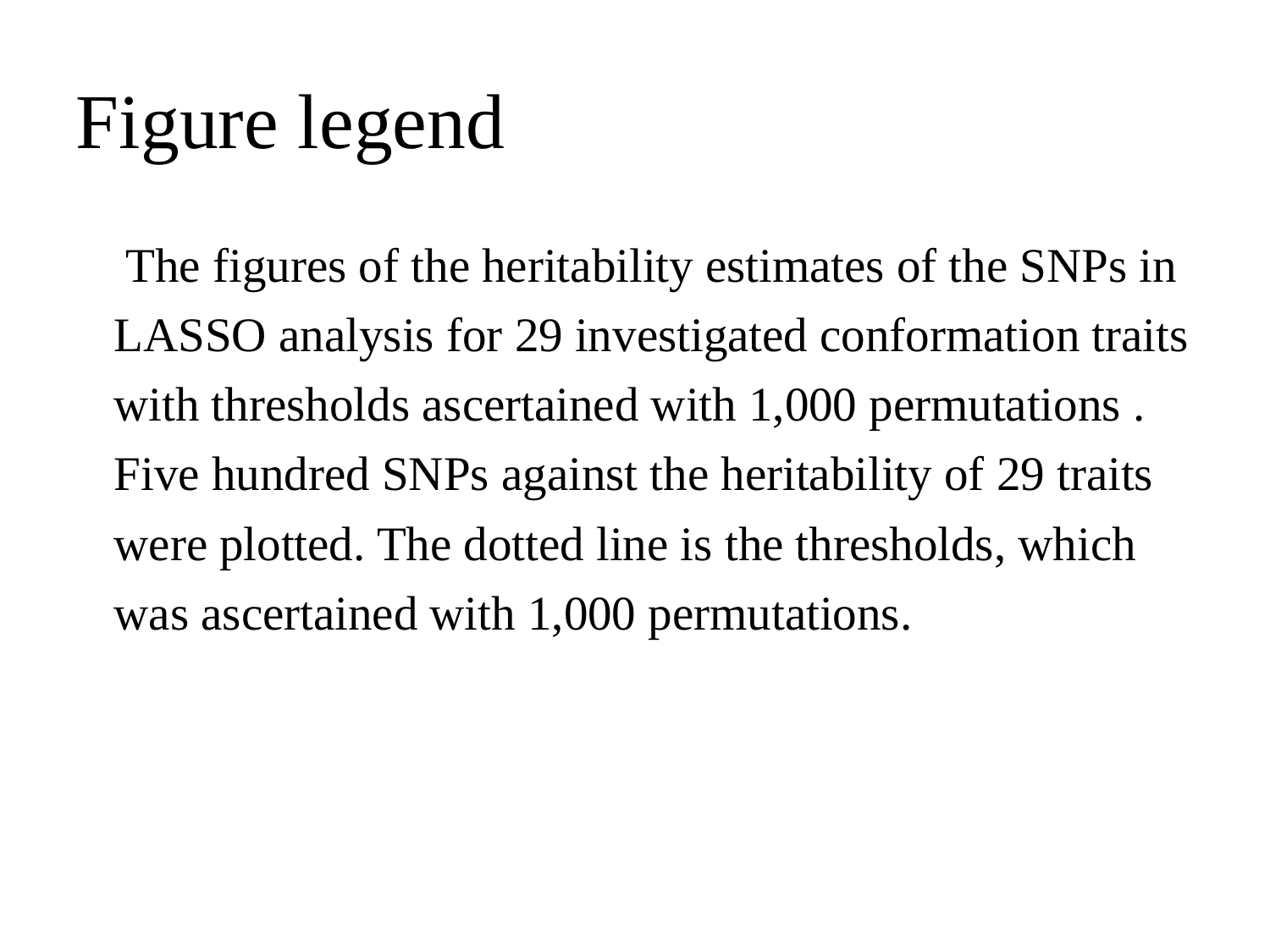

# Figure legend
 The figures of the heritability estimates of the SNPs in LASSO analysis for 29 investigated conformation traits with thresholds ascertained with 1,000 permutations . Five hundred SNPs against the heritability of 29 traits were plotted. The dotted line is the thresholds, which was ascertained with 1,000 permutations.

## Slide 3
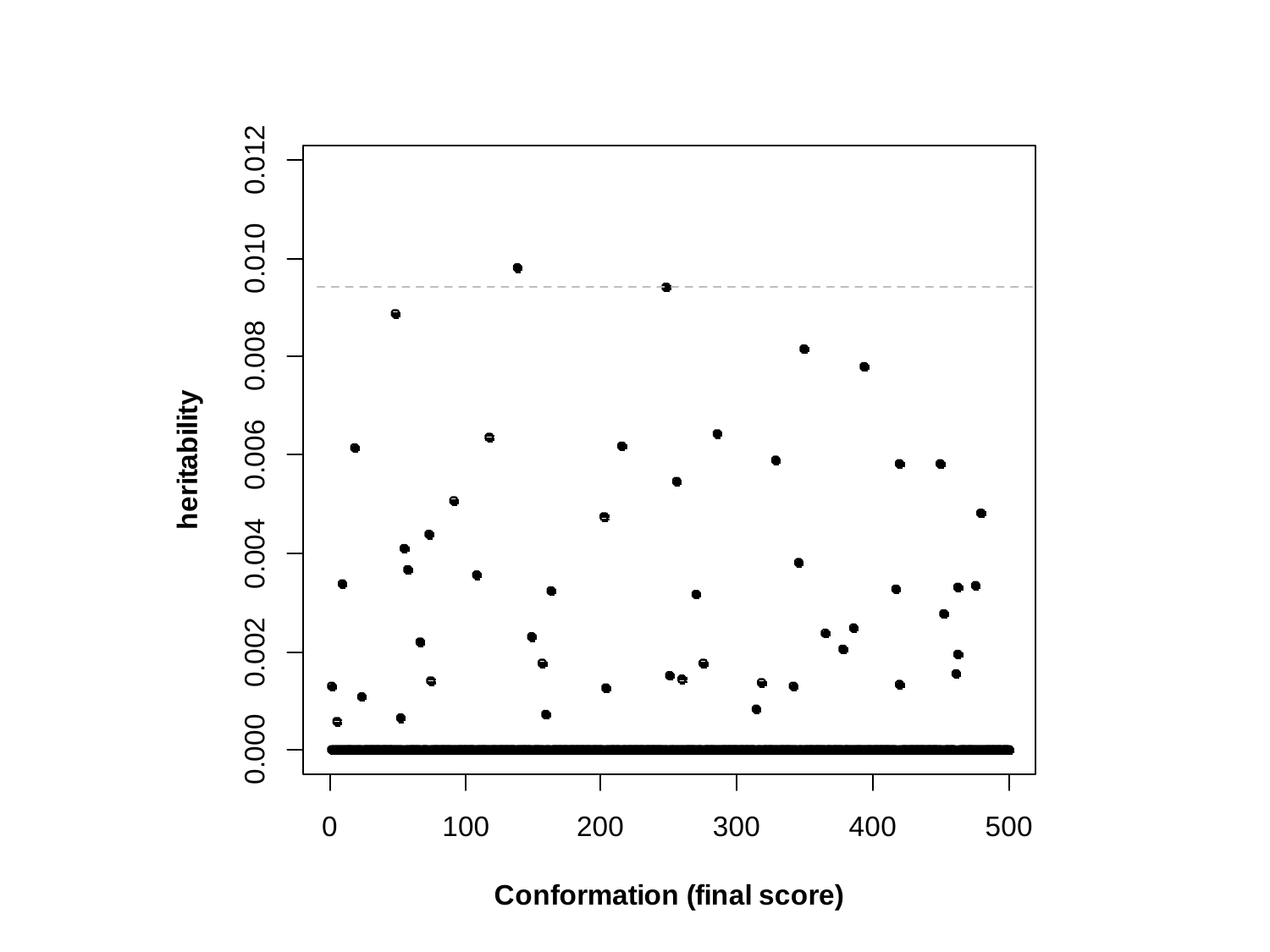

## Slide 4
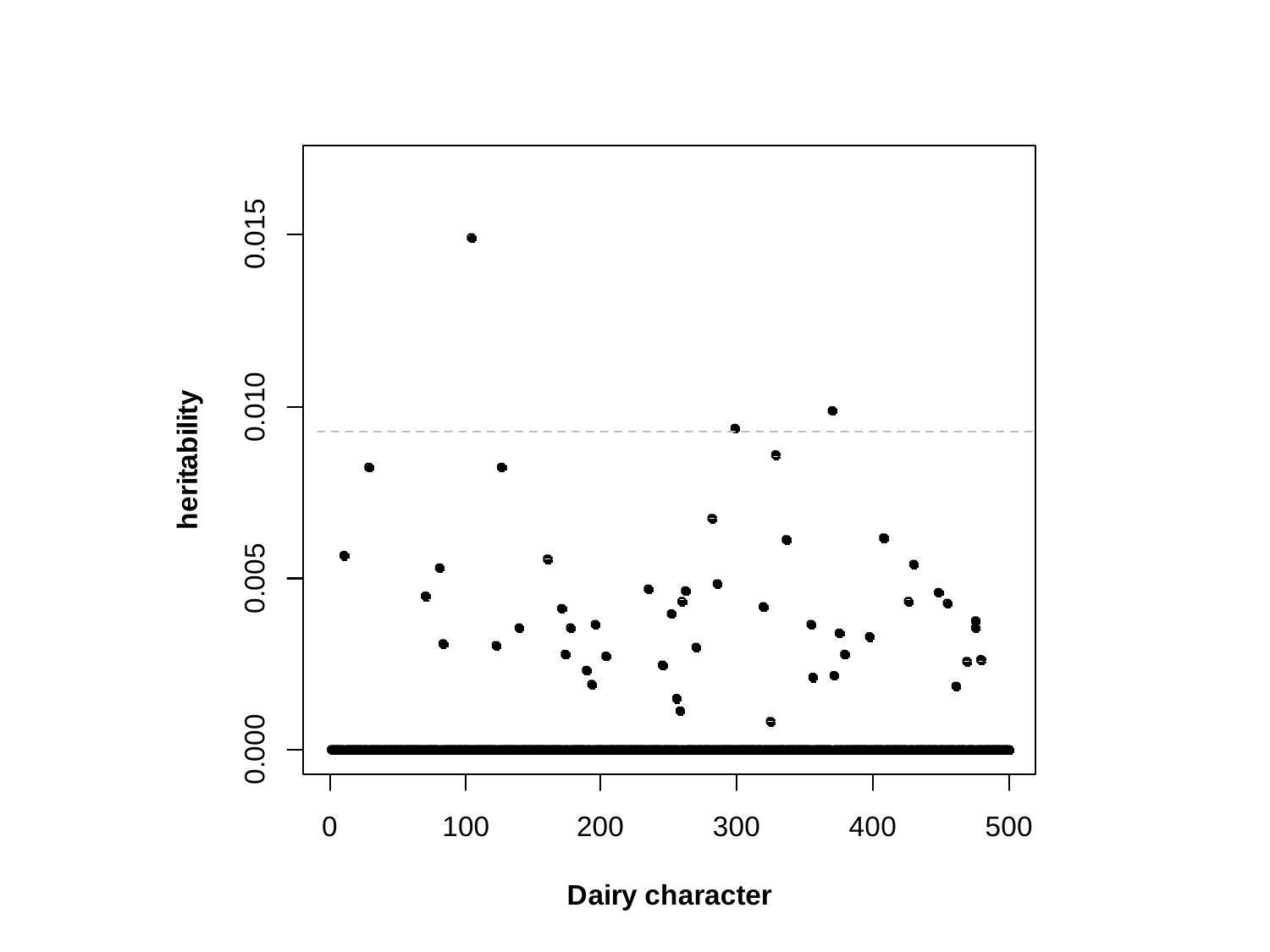

## Slide 5
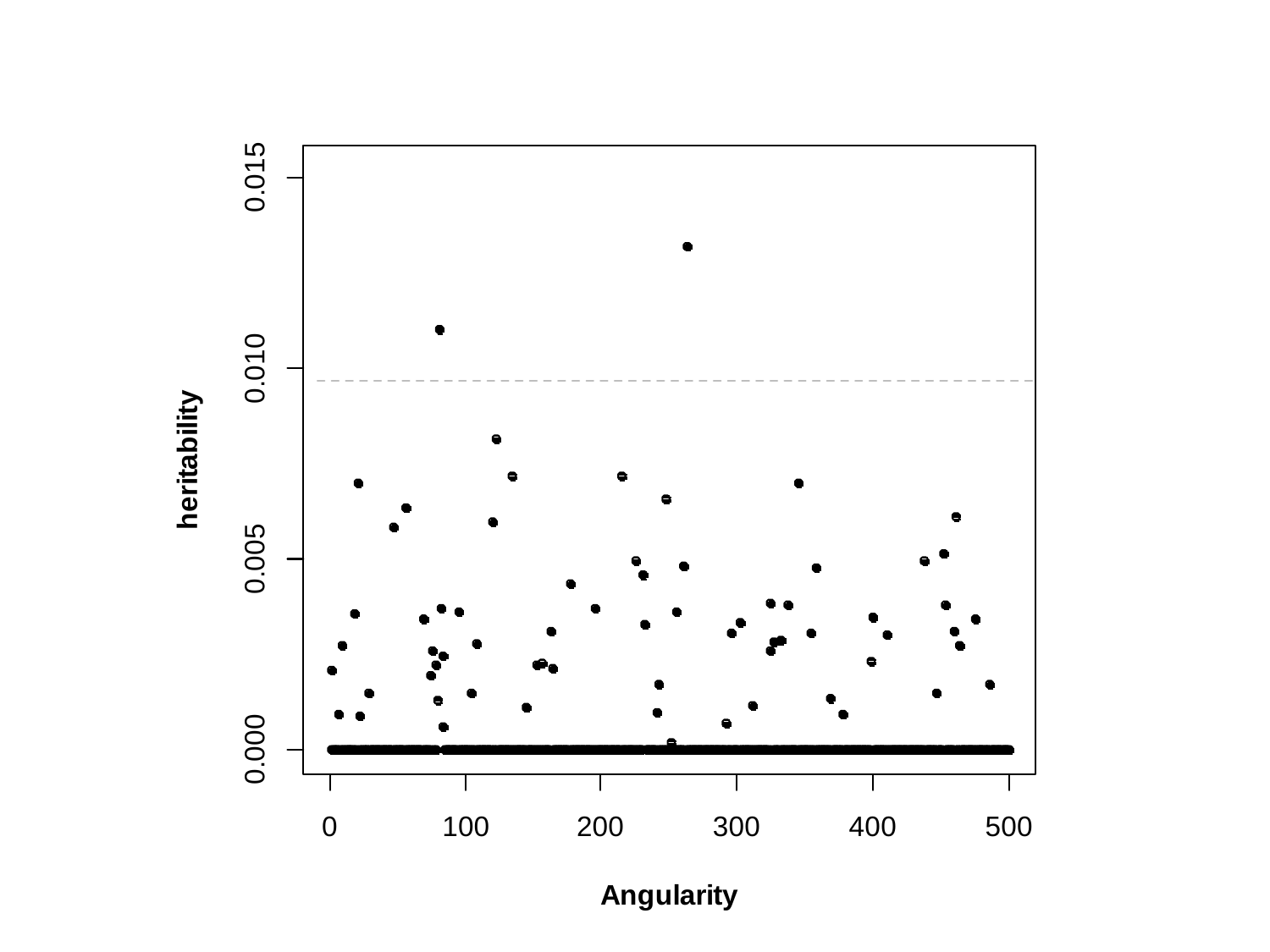

## Slide 6
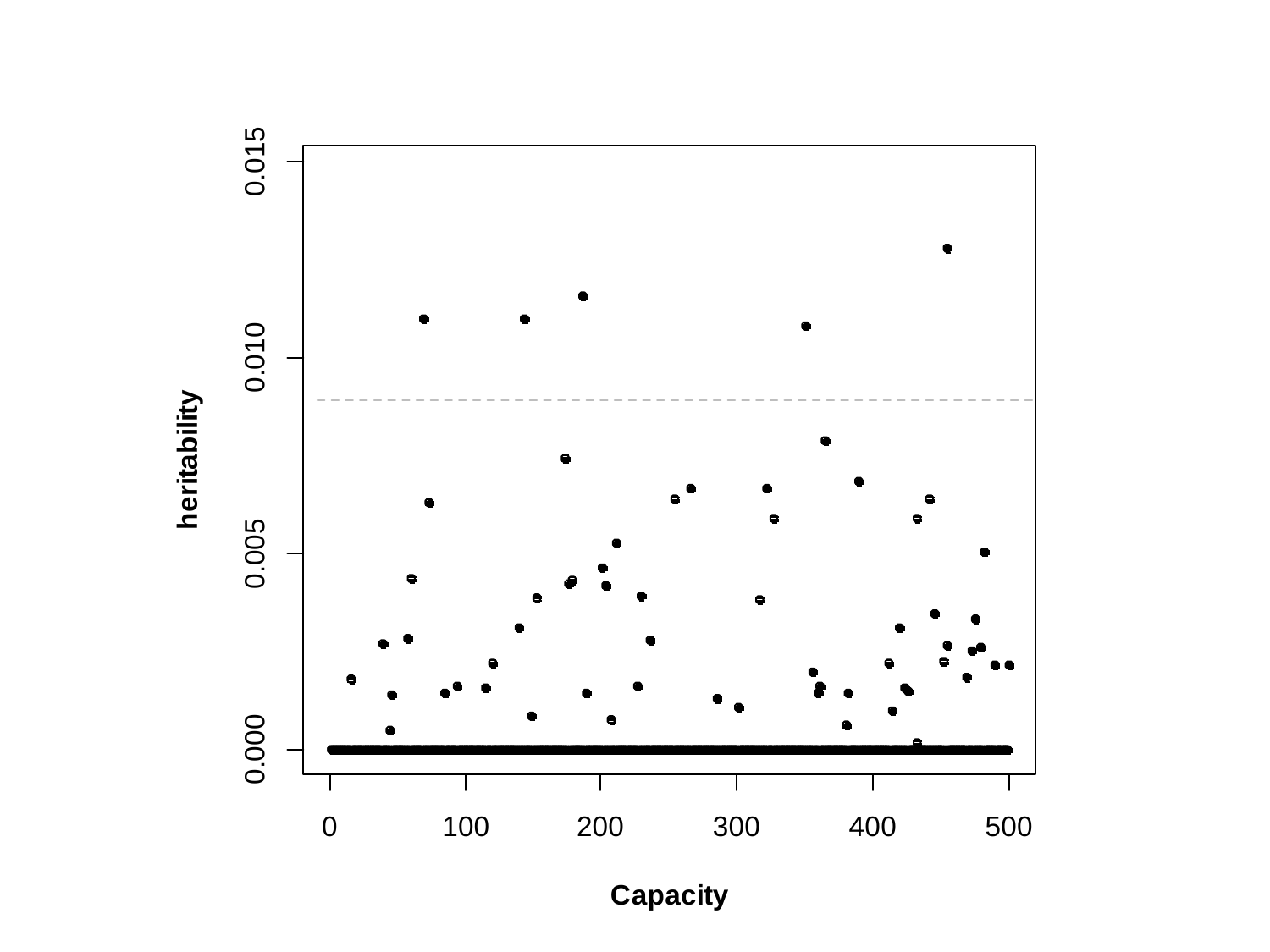

## Slide 7
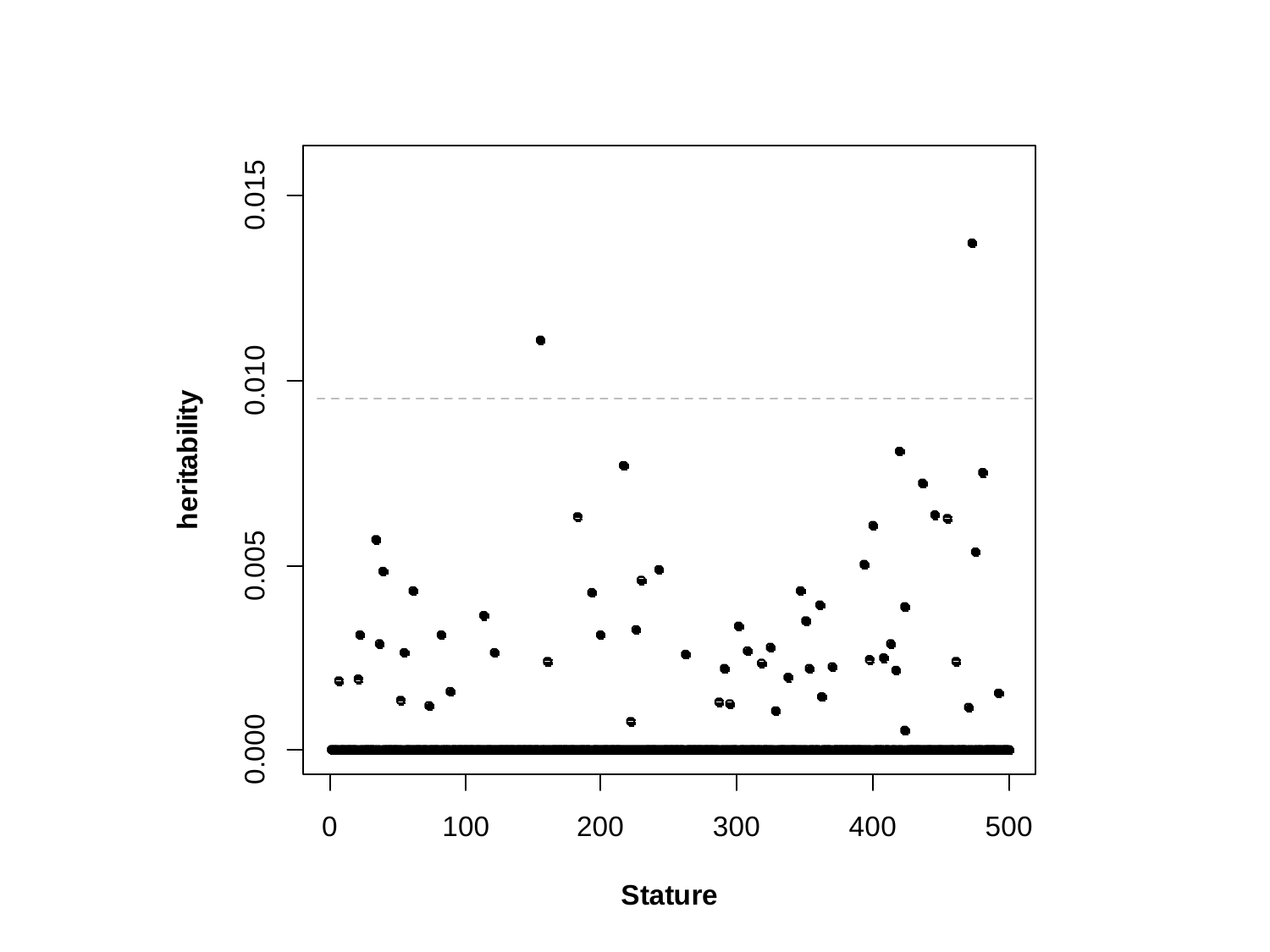

## Slide 8
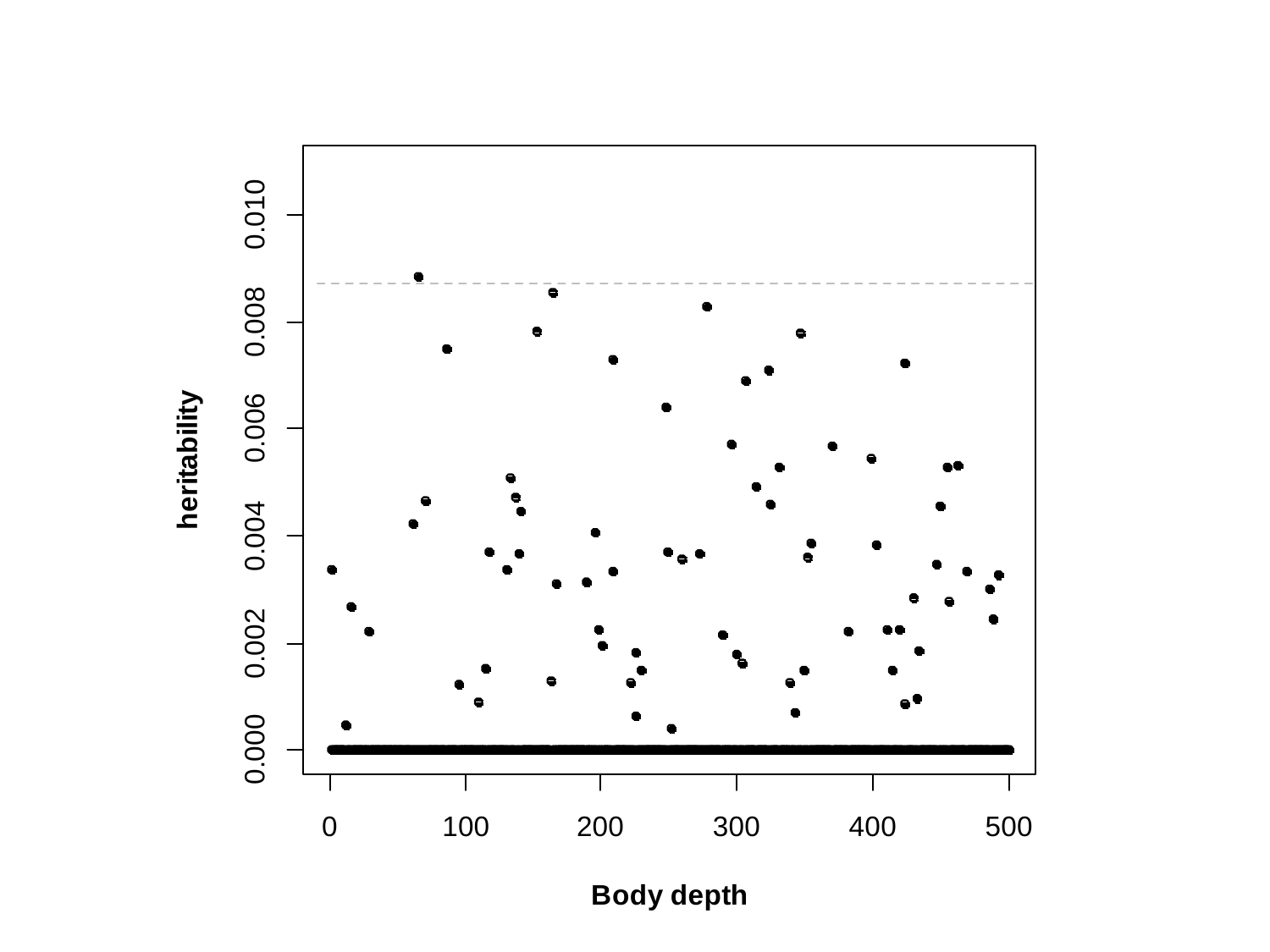

## Slide 9
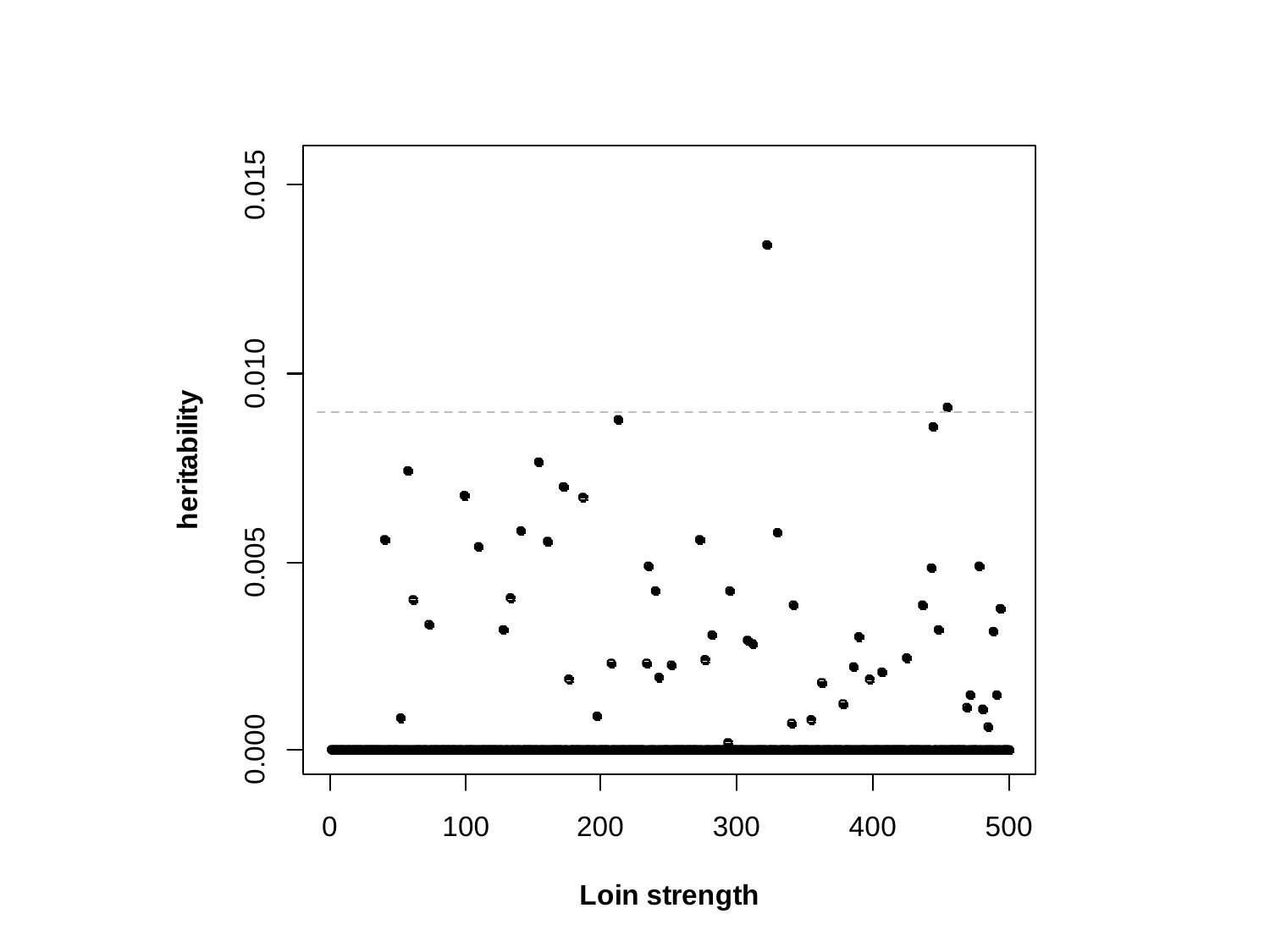

## Slide 10
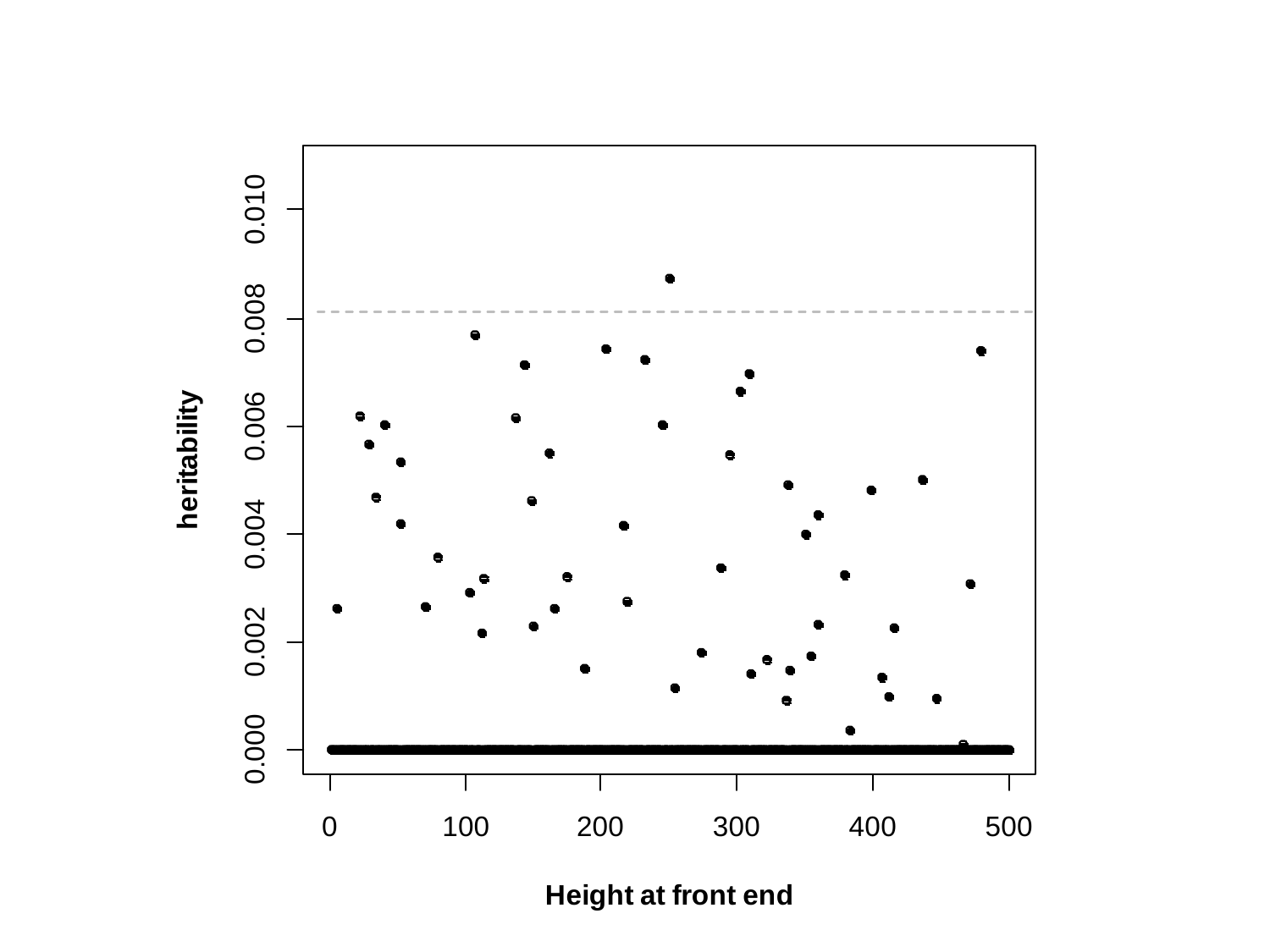

## Slide 11
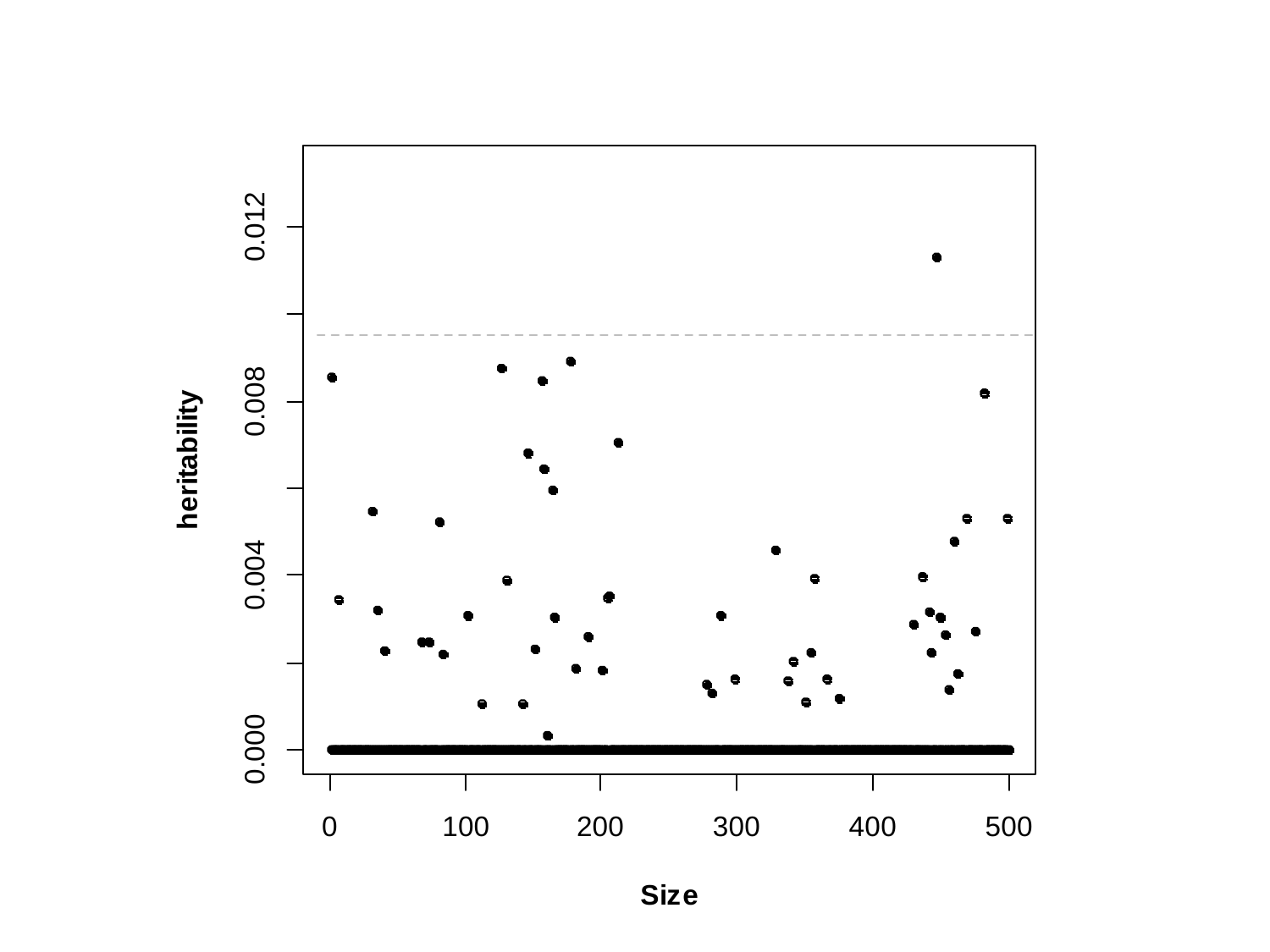

## Slide 12
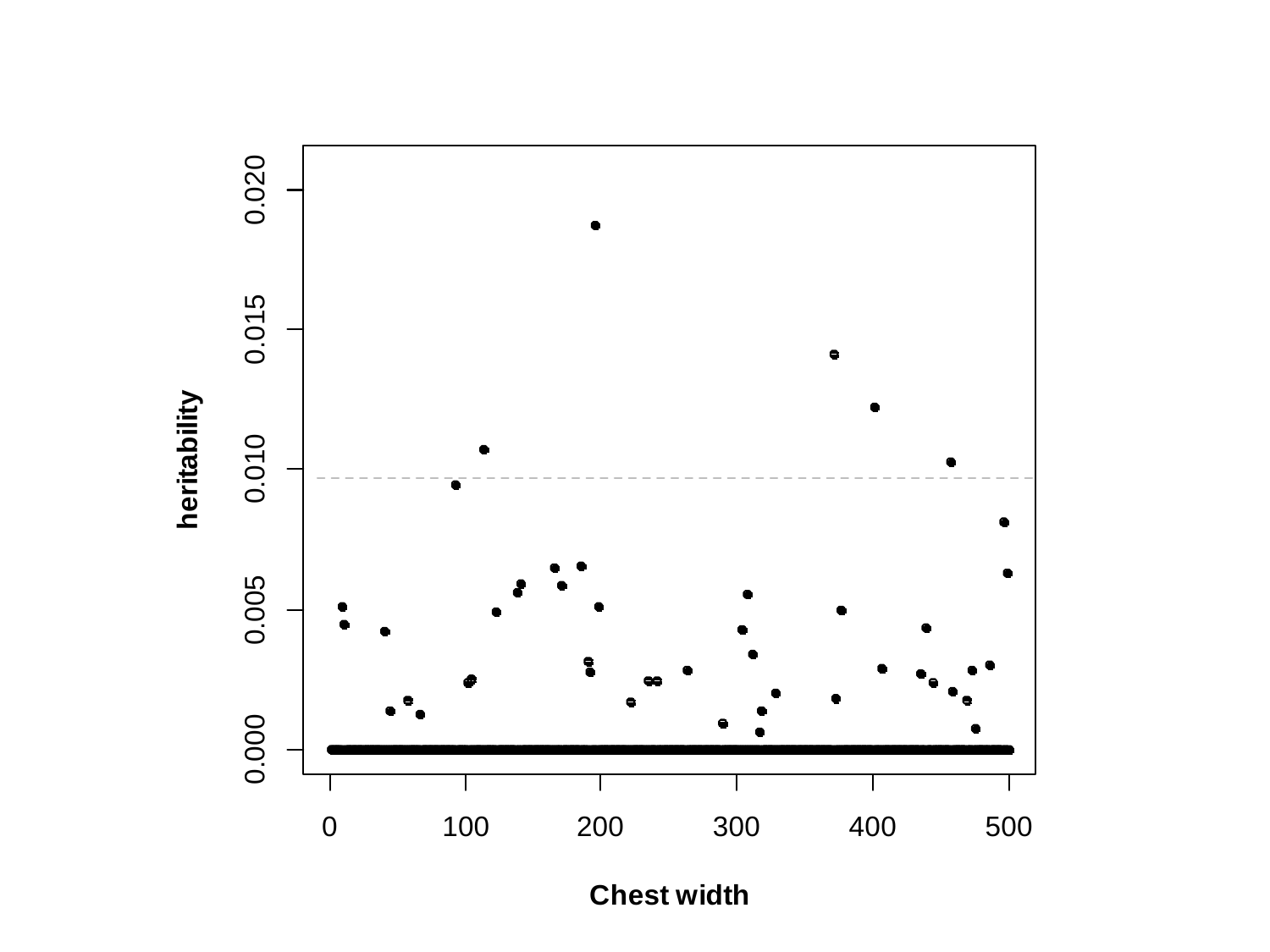

## Slide 13
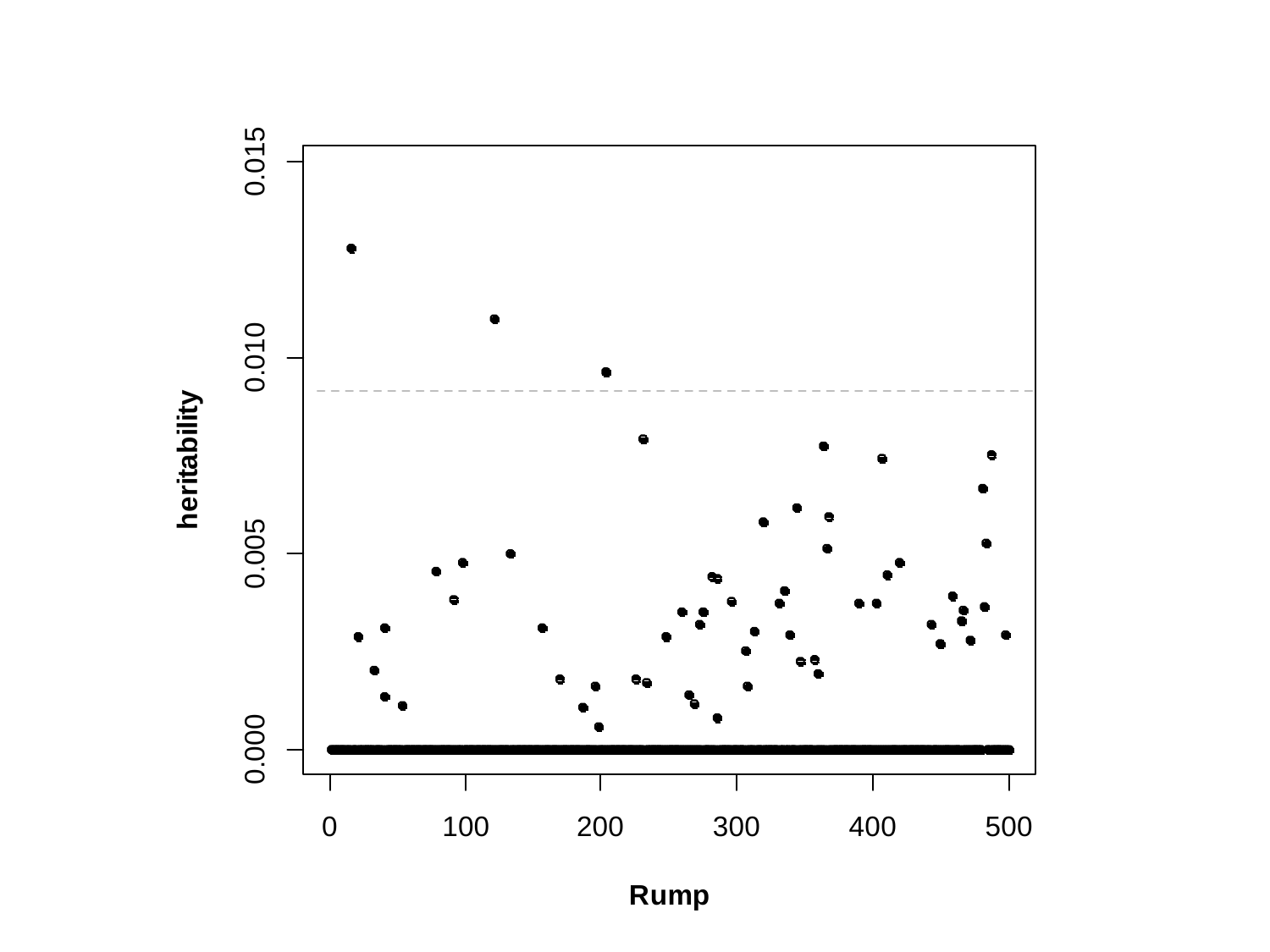

## Slide 14
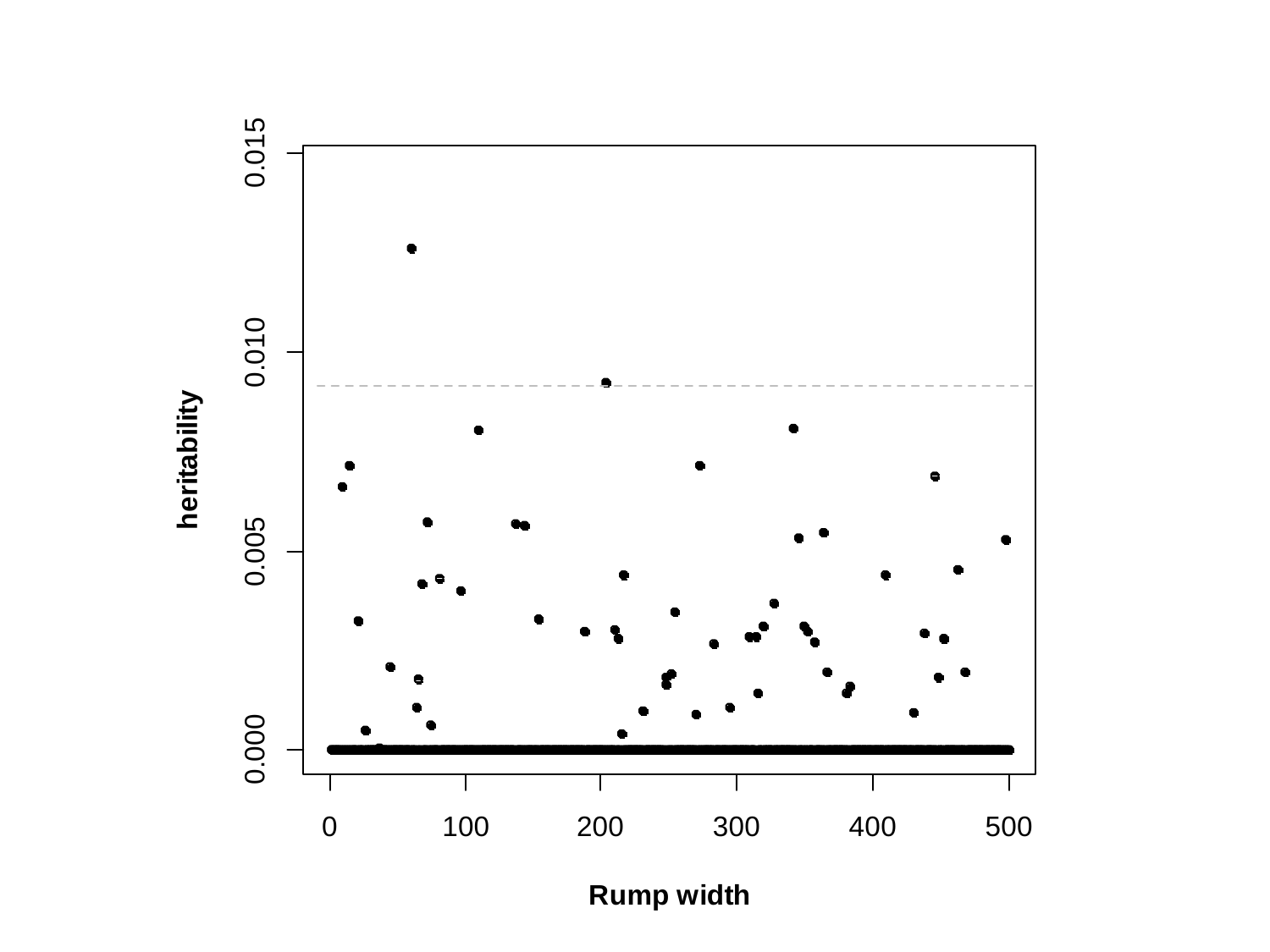

## Slide 15
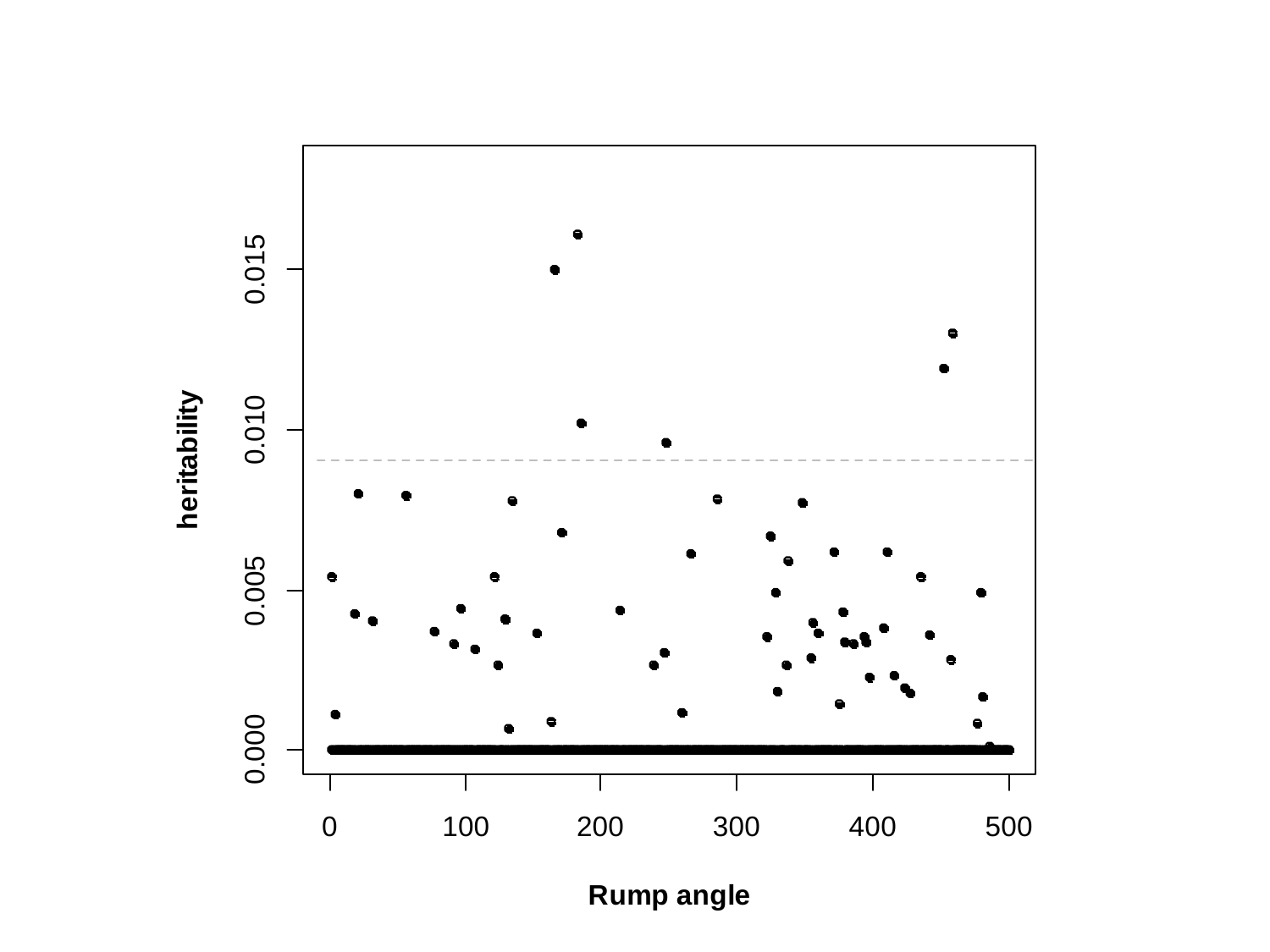

## Slide 16
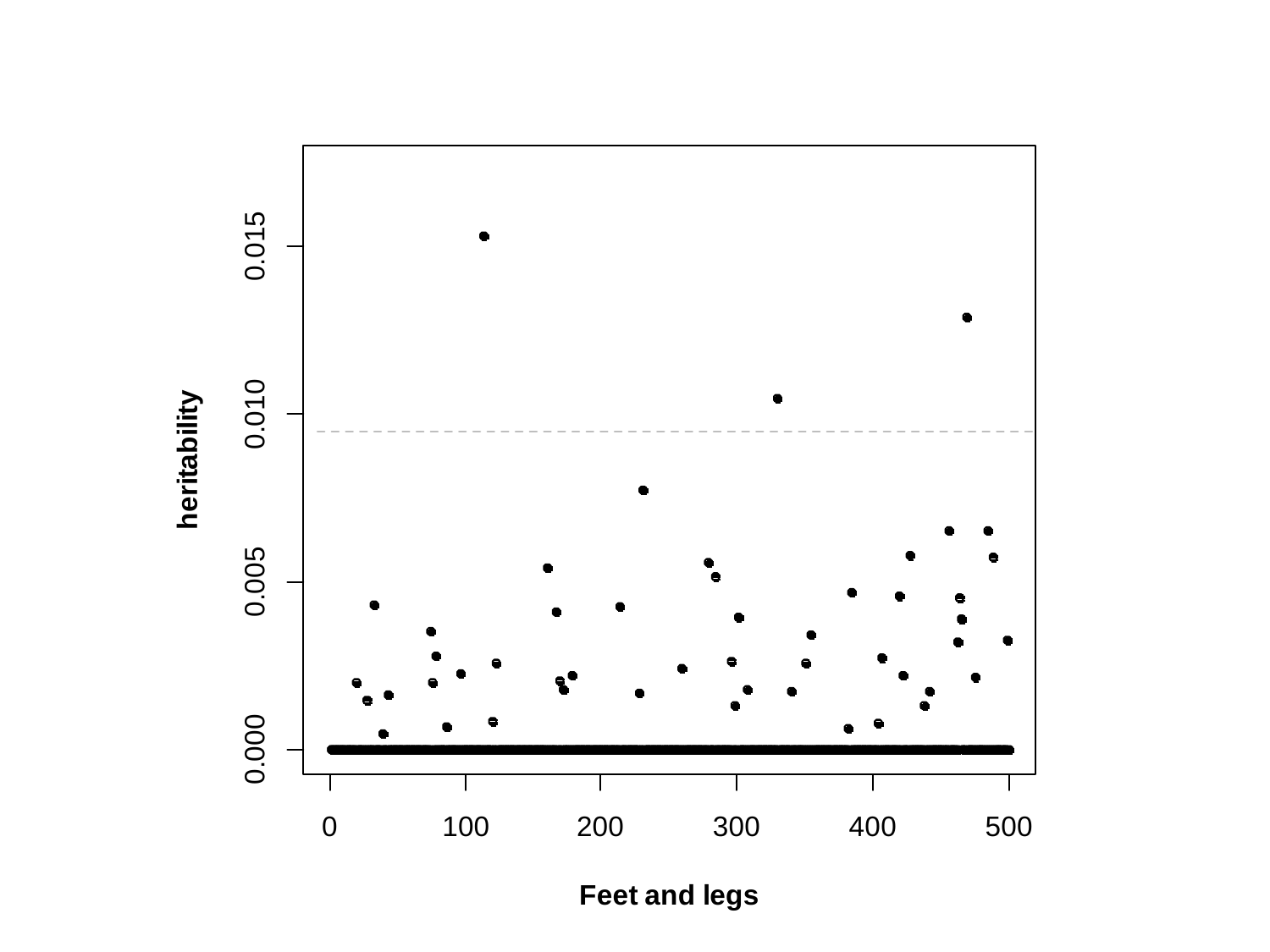

## Slide 17
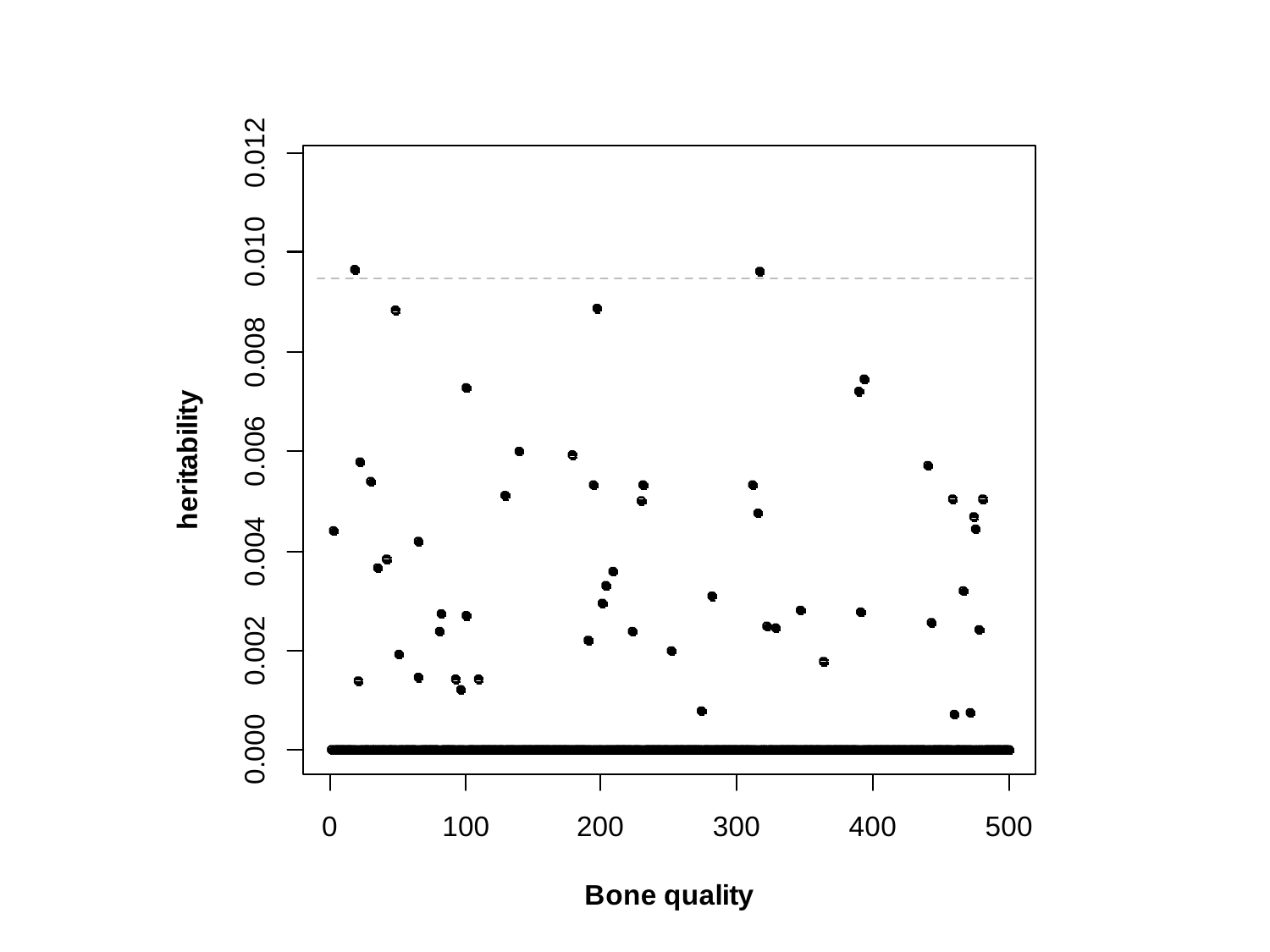

## Slide 18
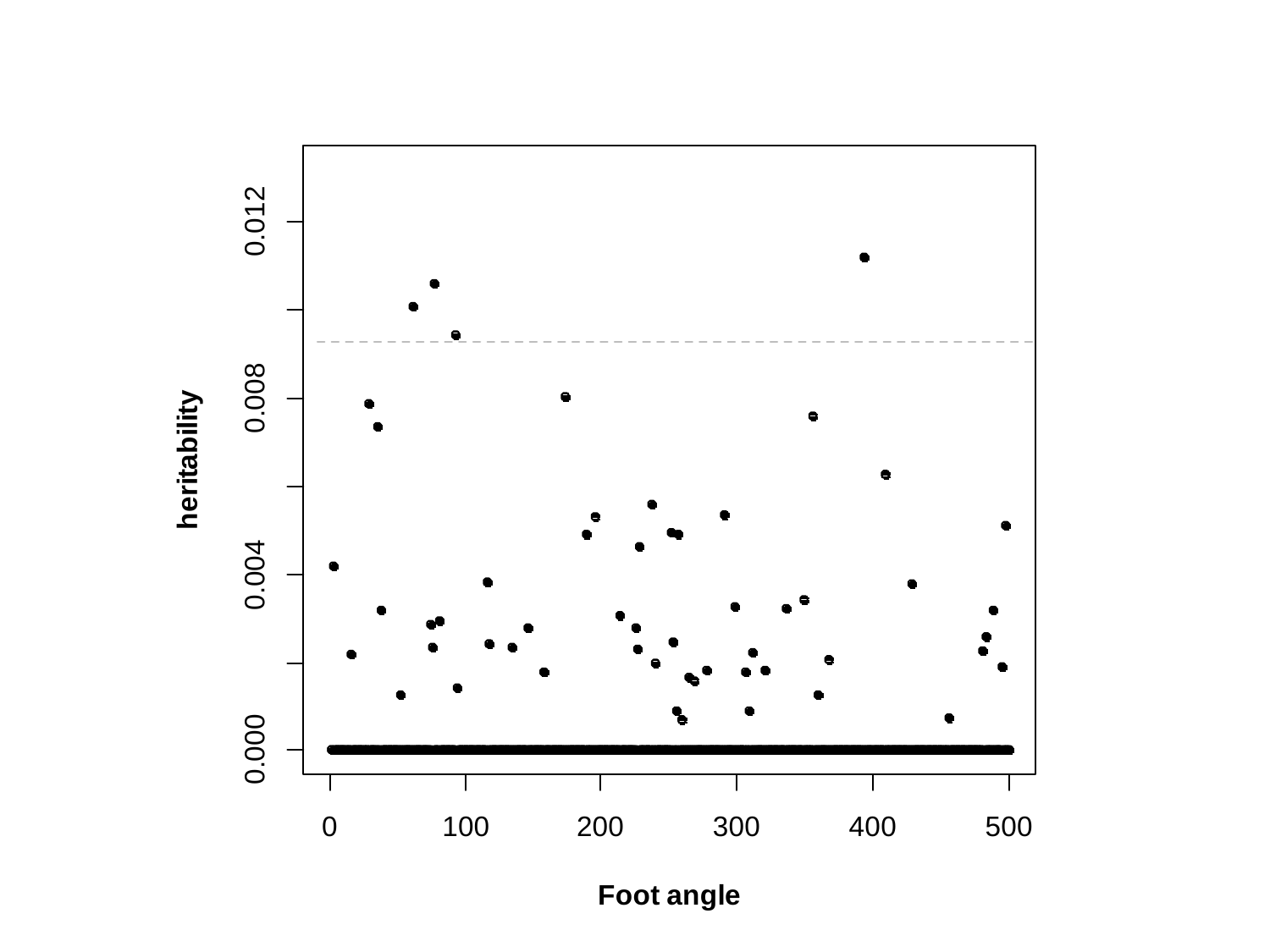

## Slide 19
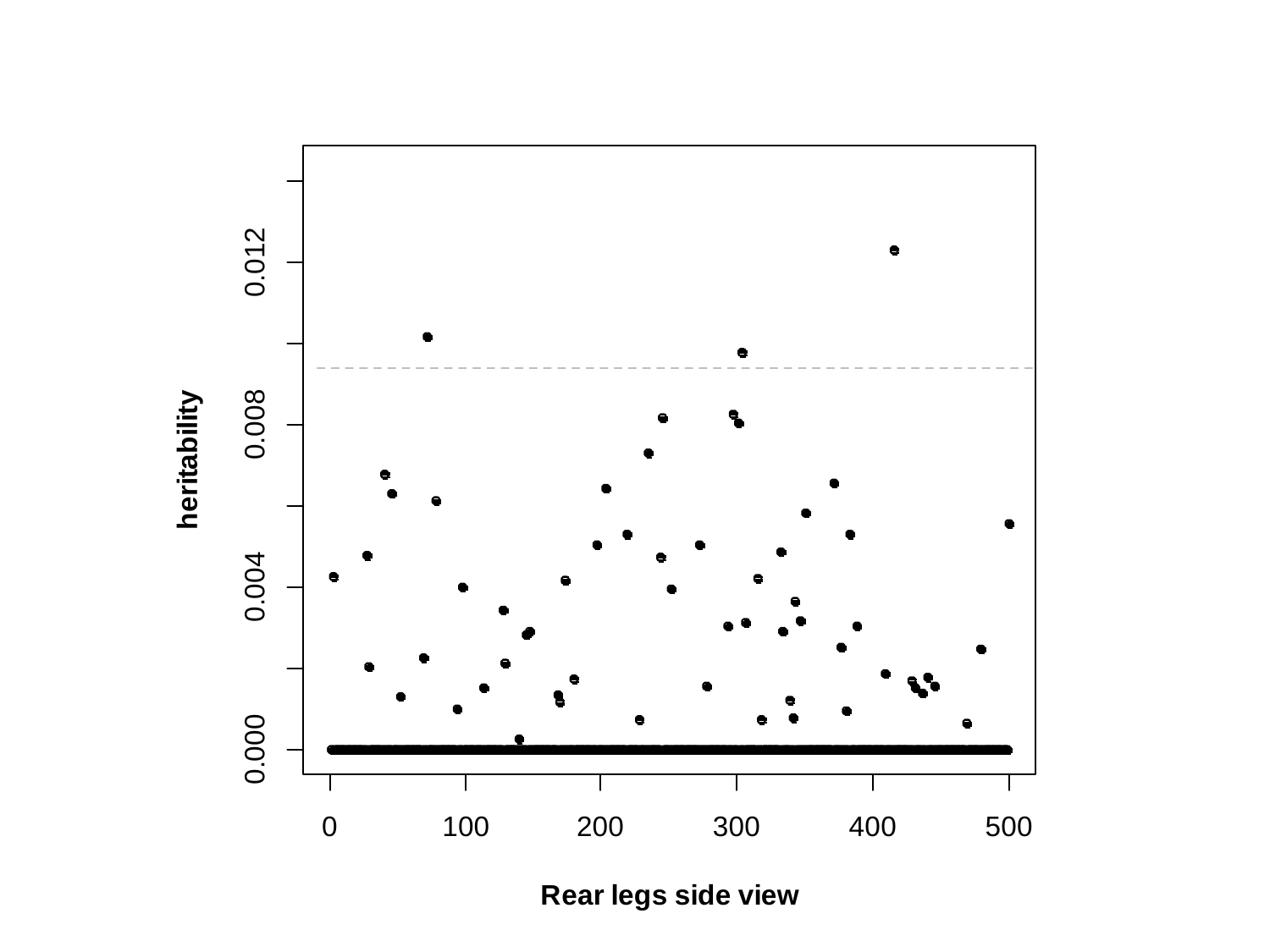

## Slide 20
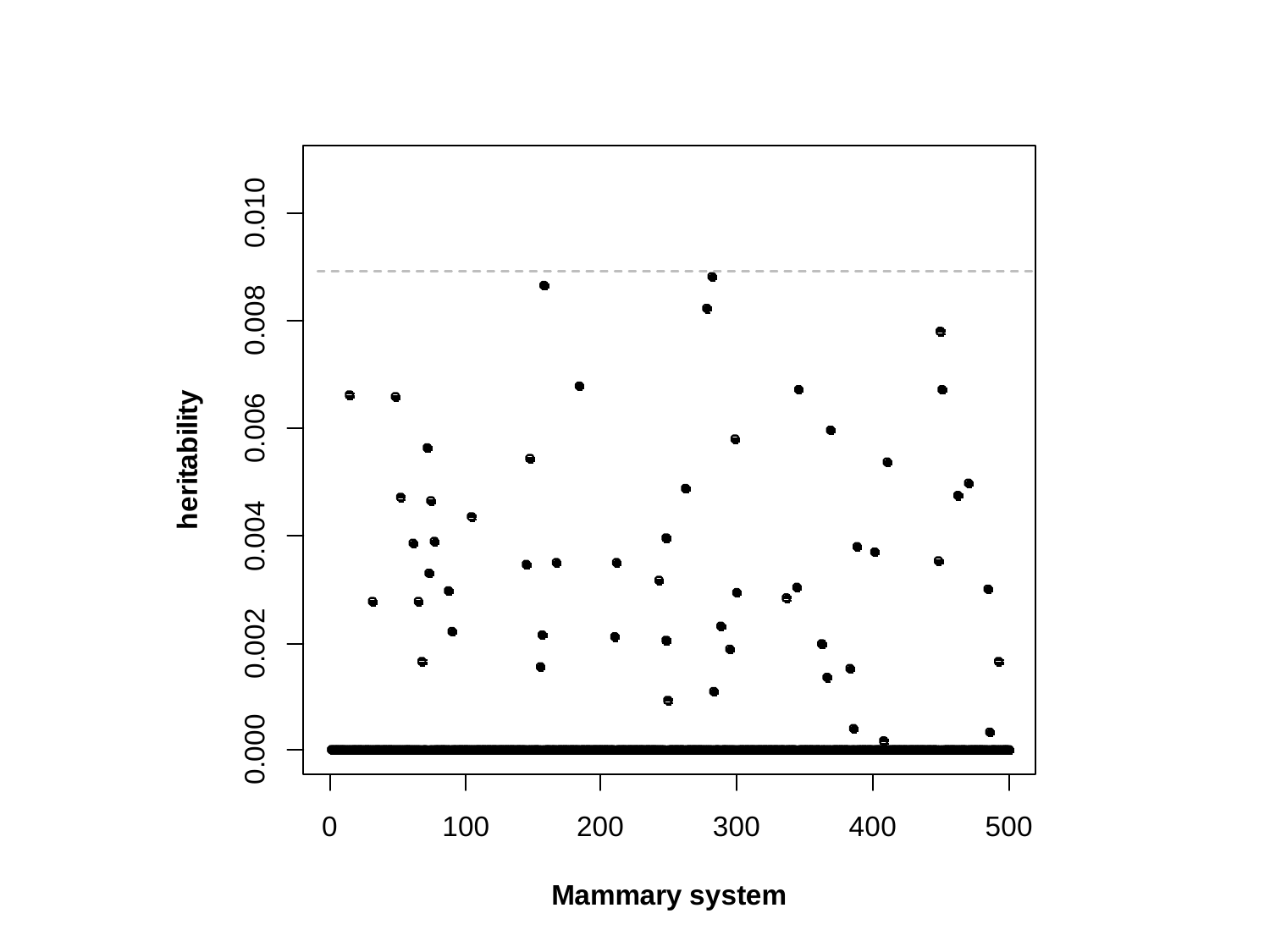

## Slide 21
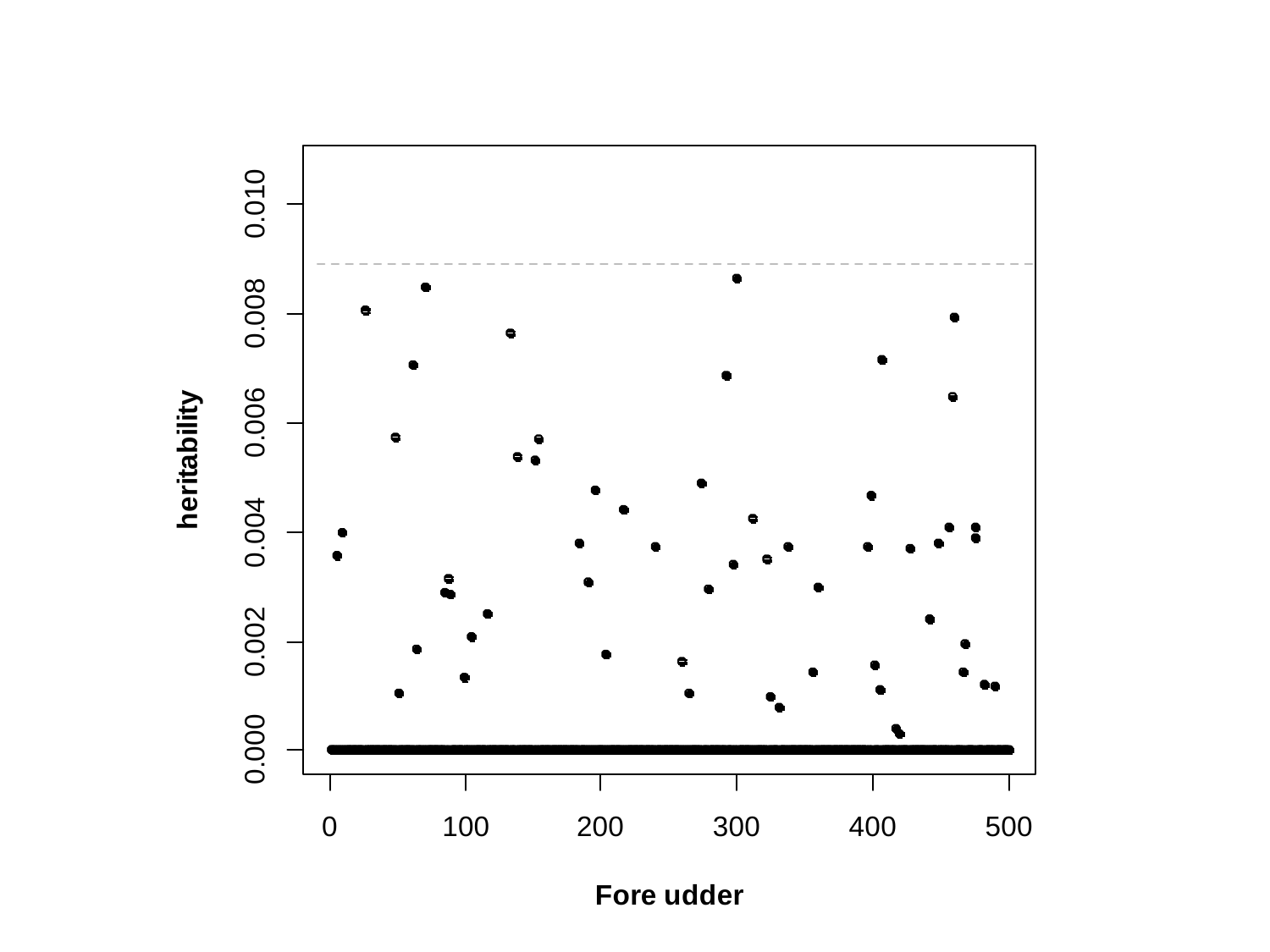

## Slide 22
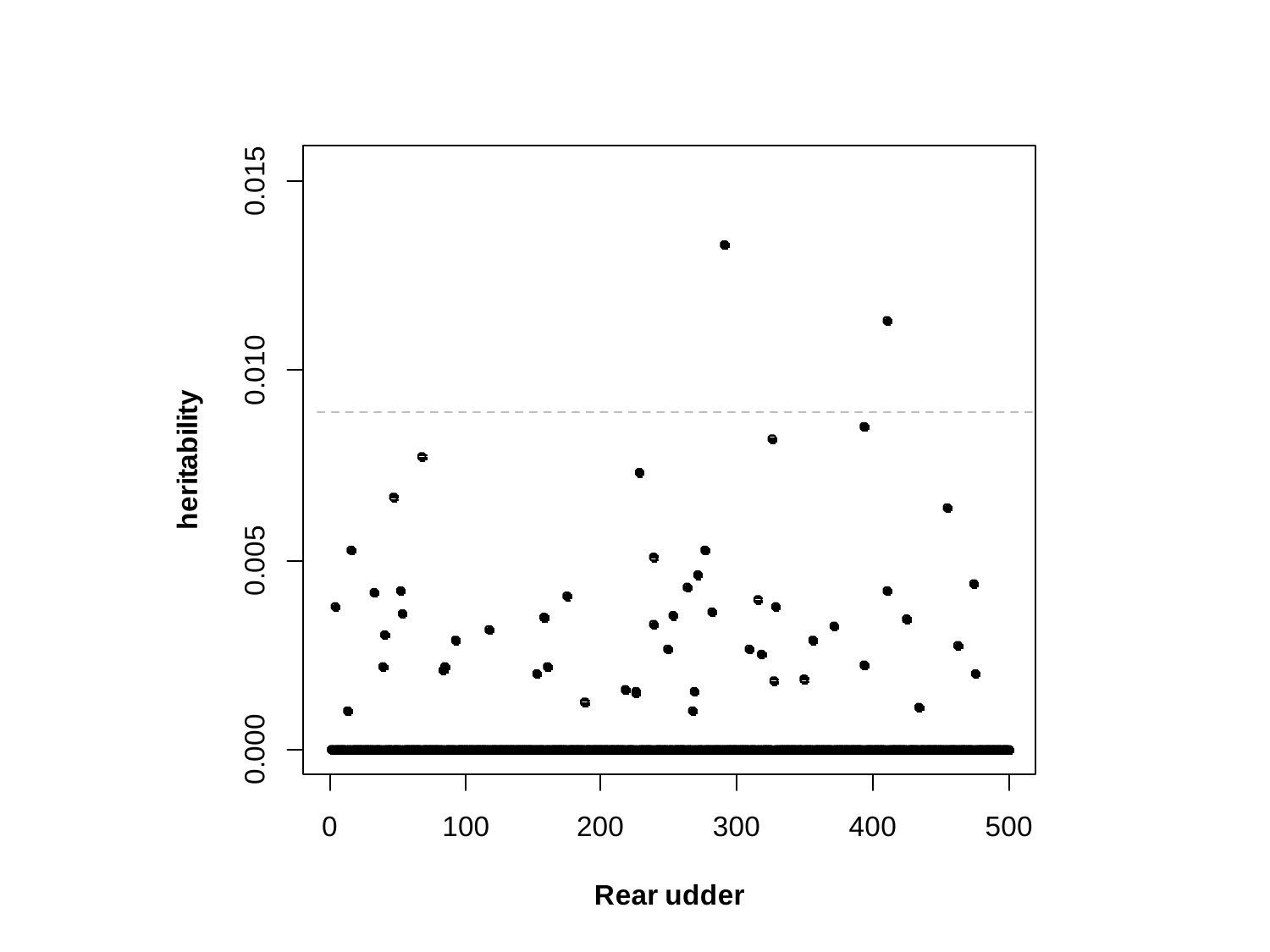

## Slide 23
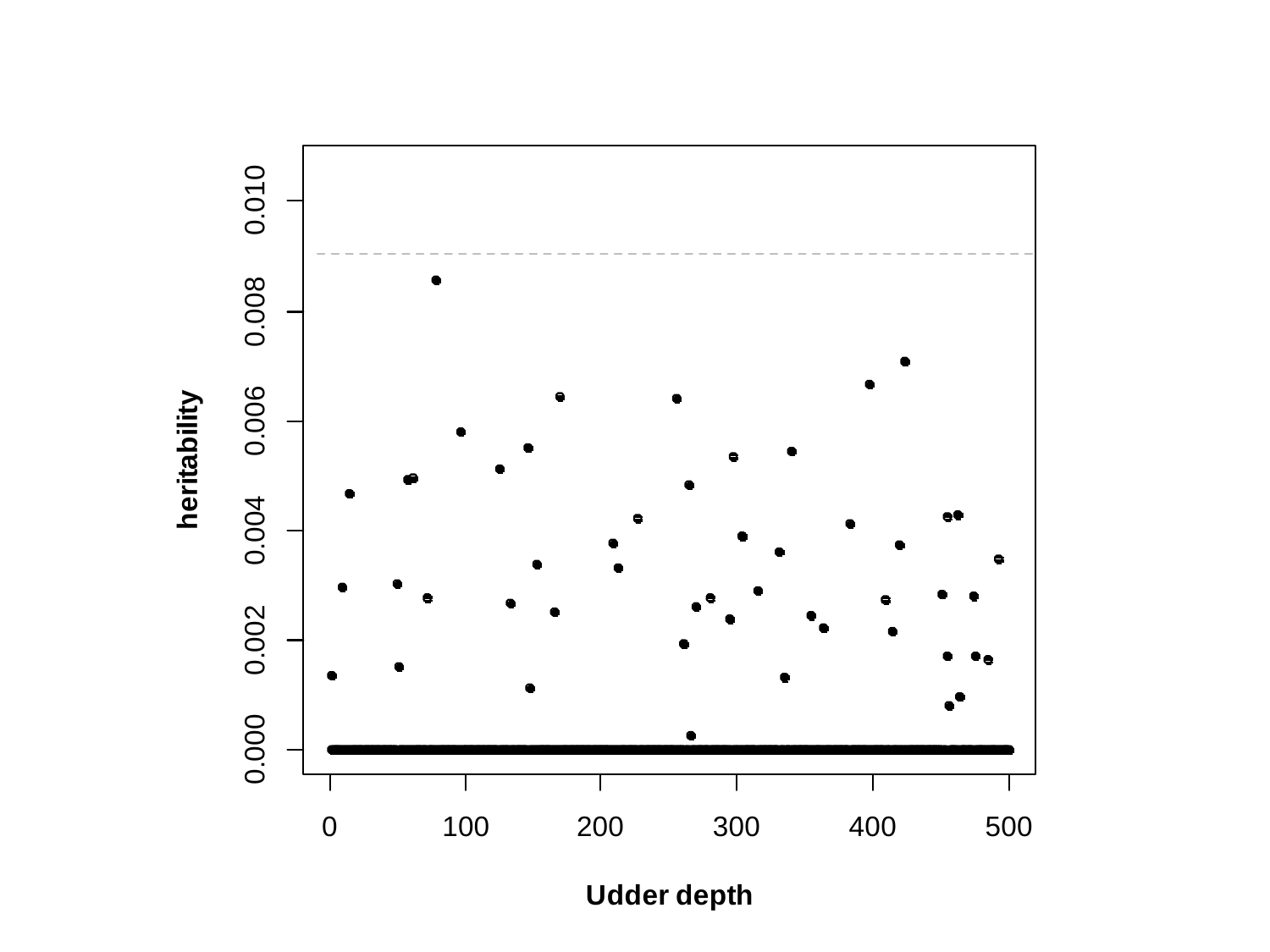

## Slide 24
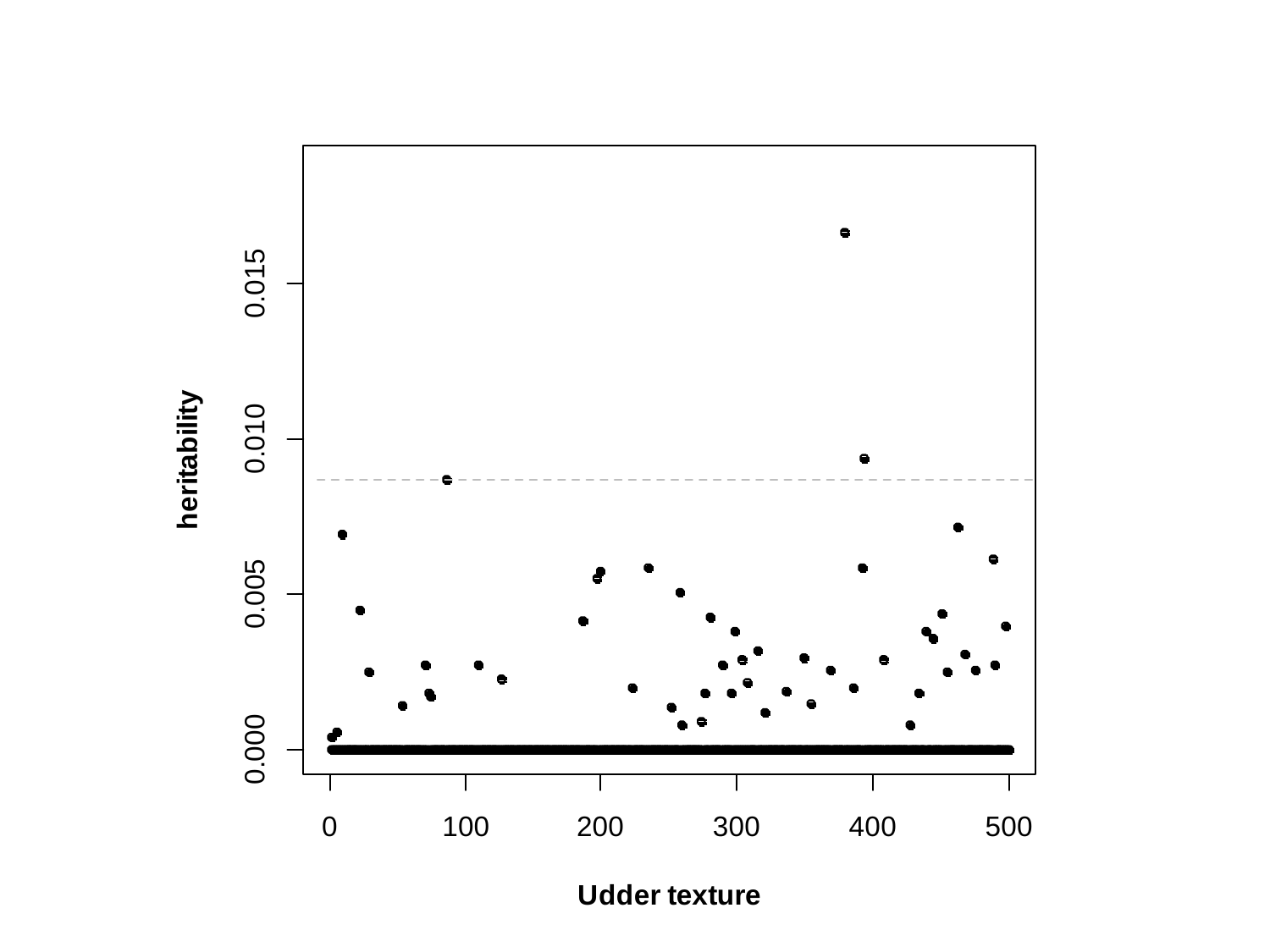

## Slide 25
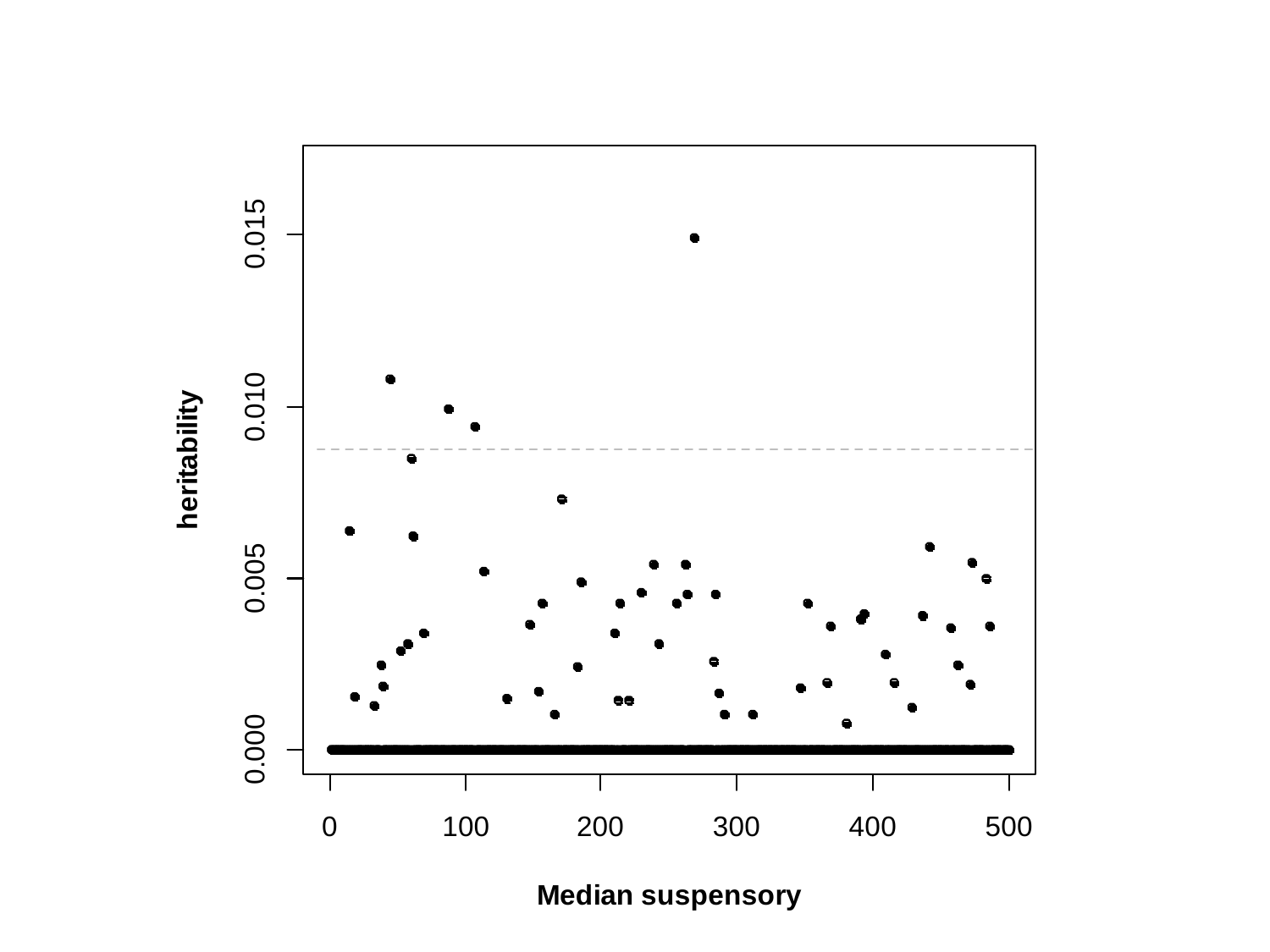

## Slide 26
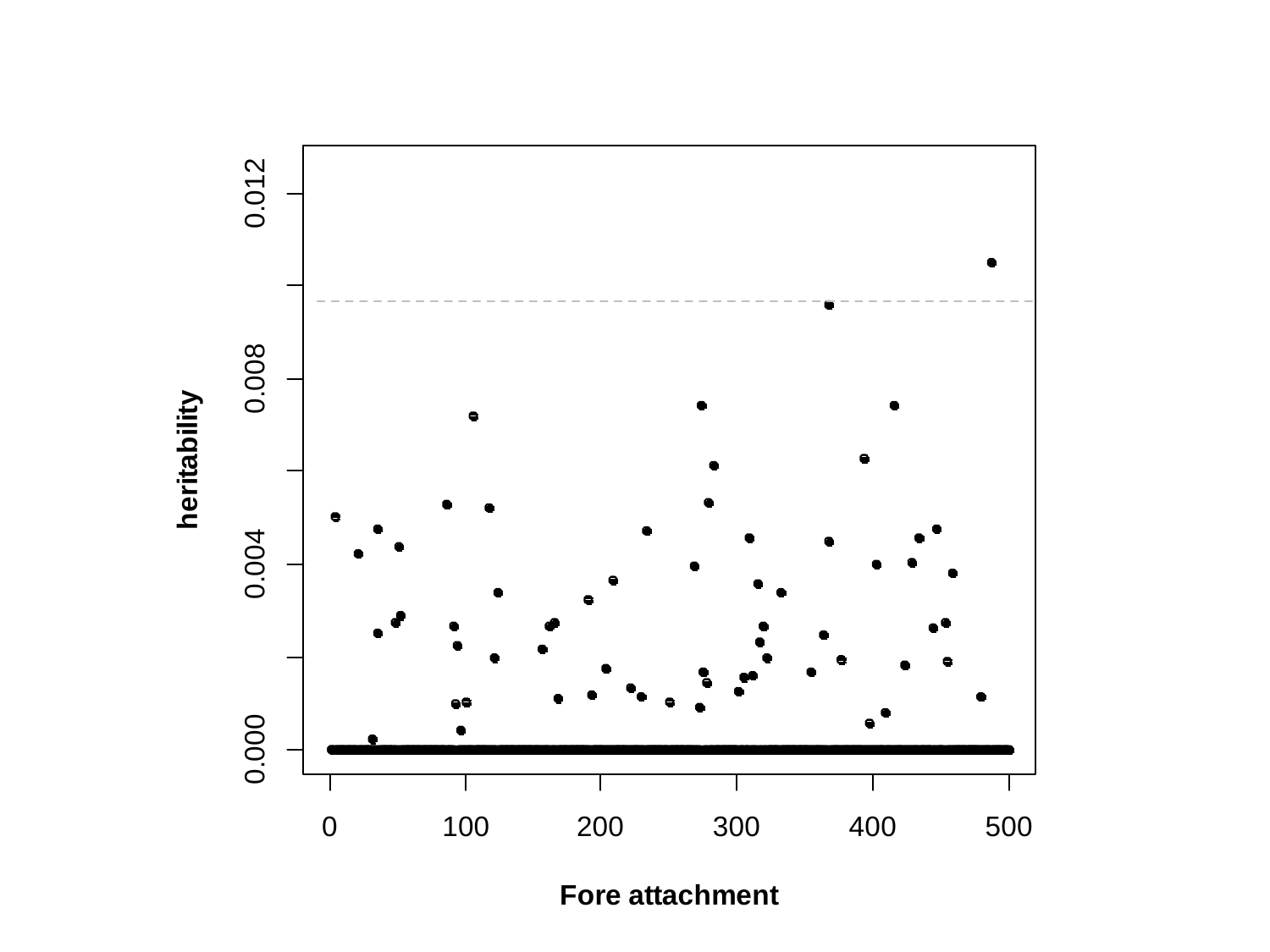

## Slide 27
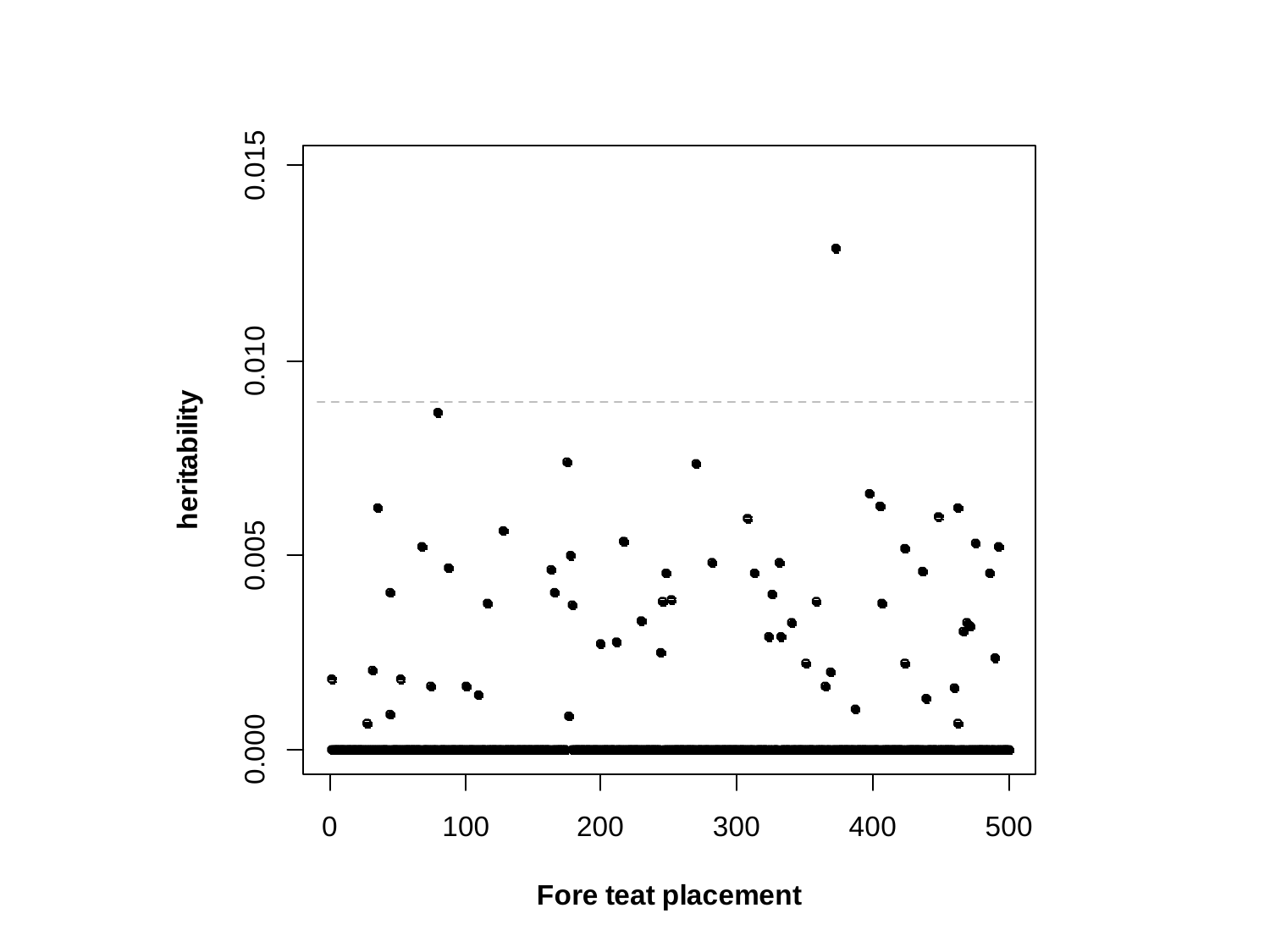

## Slide 28
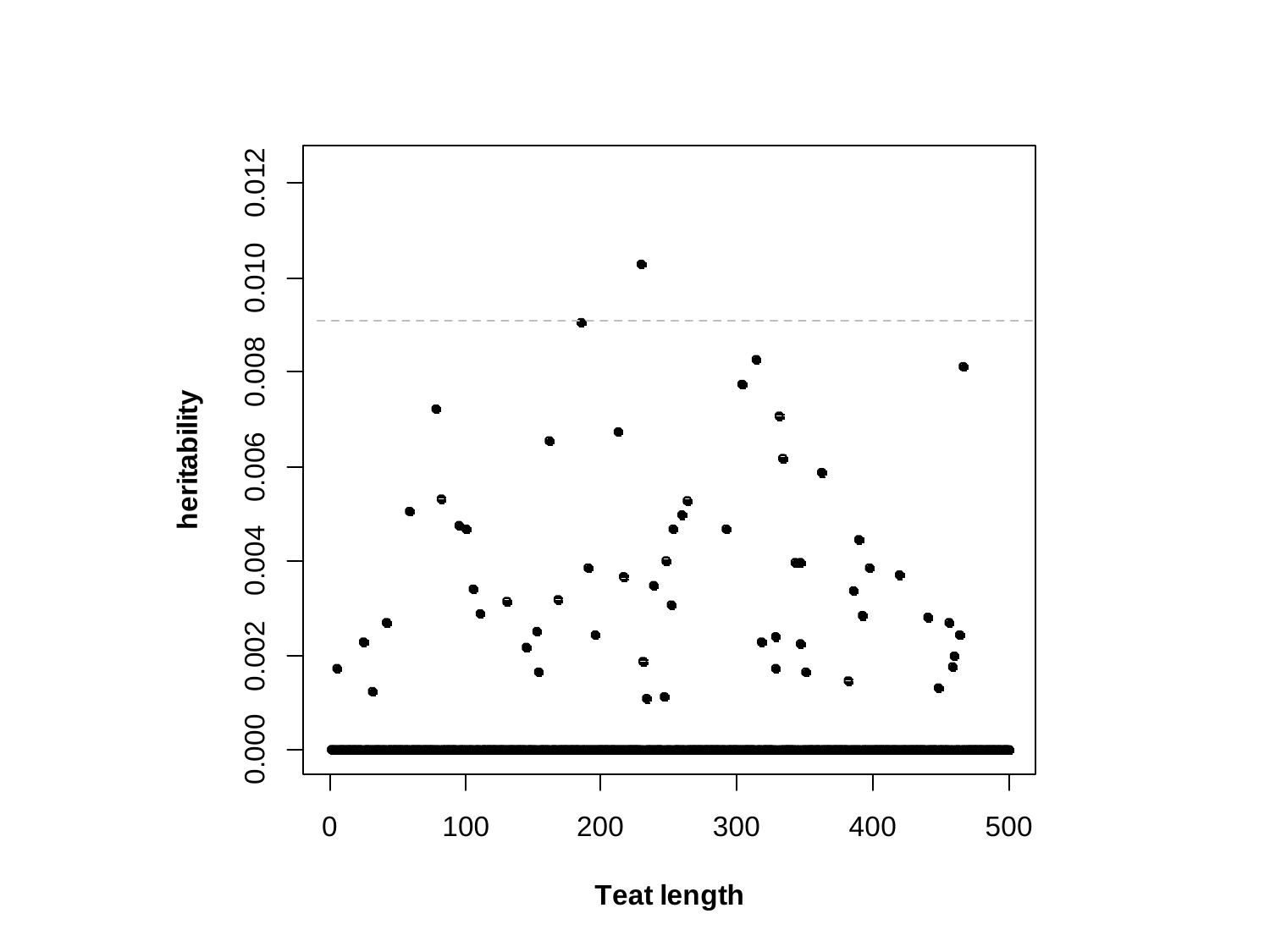

## Slide 29
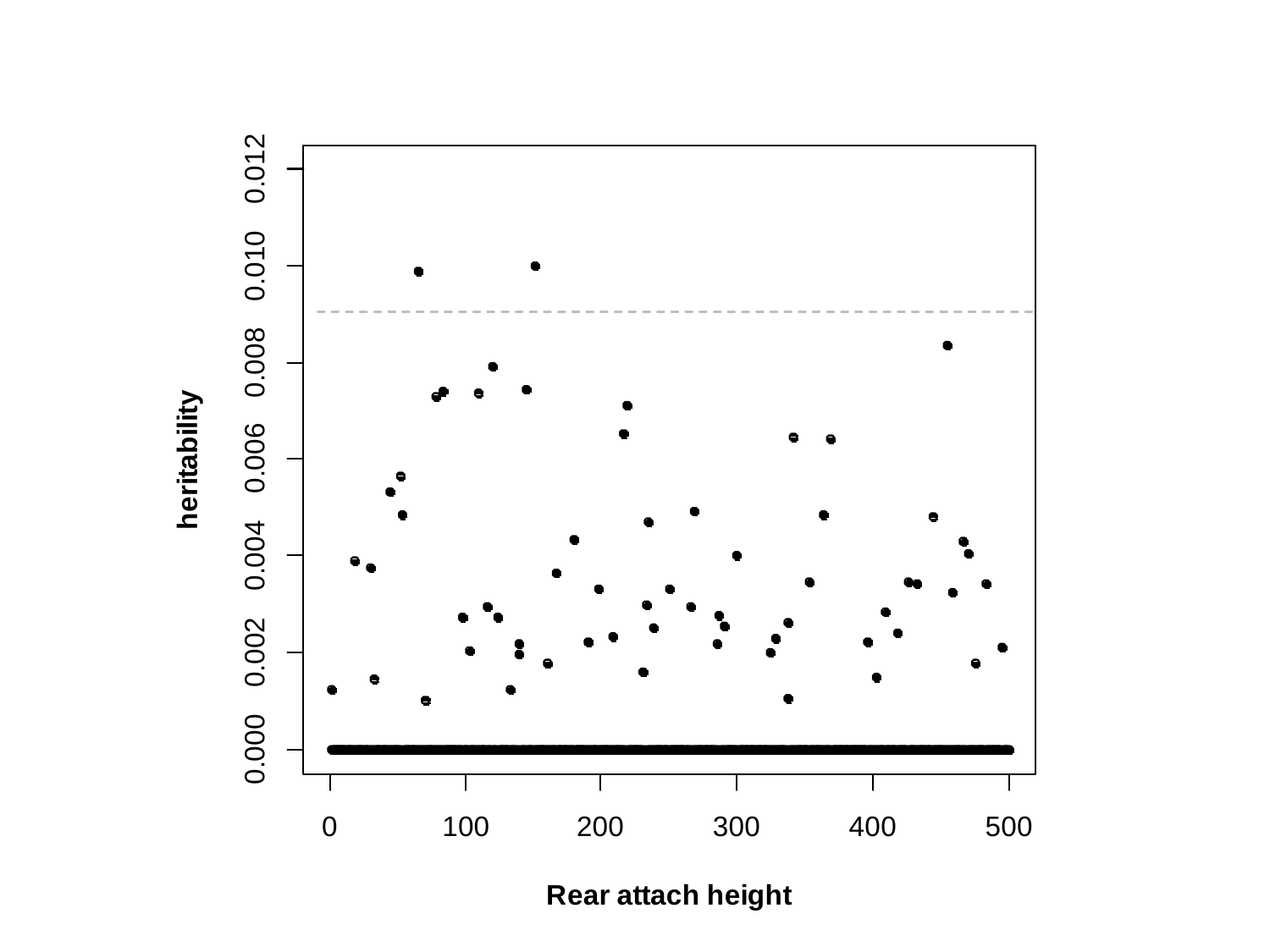

## Slide 30
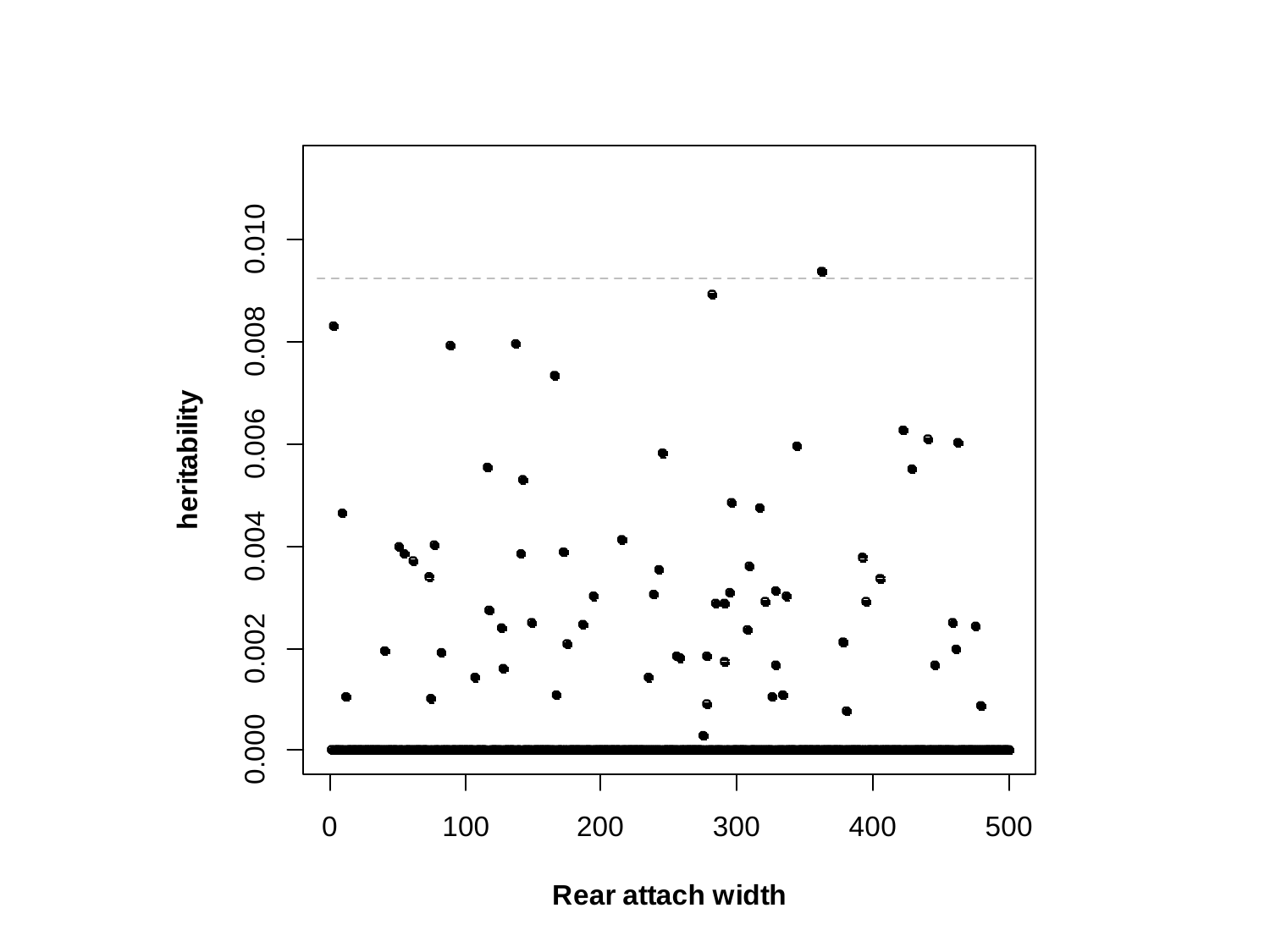

## Slide 31
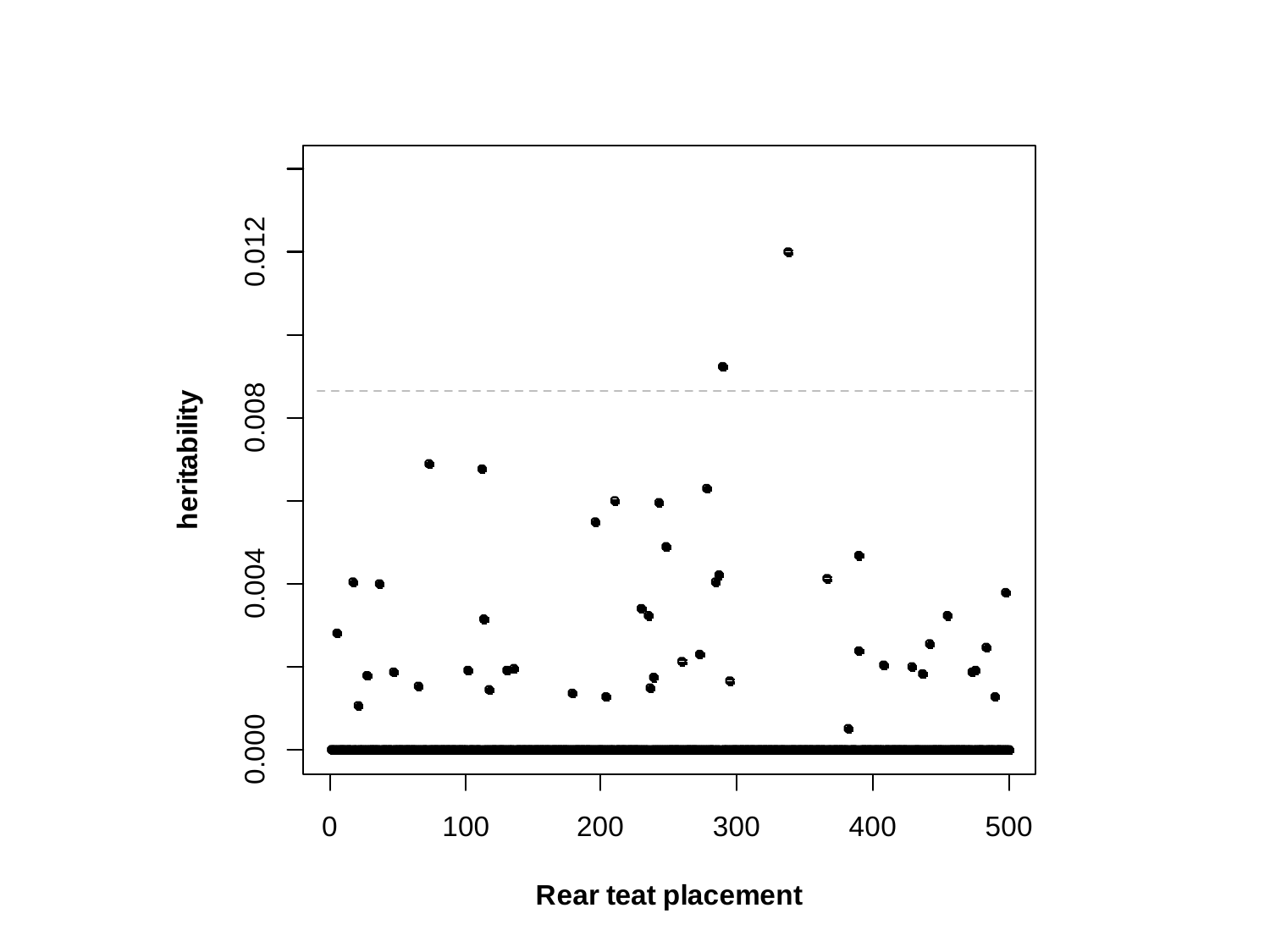

Supplement: Additional file 3 — Heritability estimates of the SNPs in LASSO analysis for the 29 investigated conformation traits. This file contains the figures of the heritability estimates of the SNPs in LASSO analysis for 29 investigated conformation traits with thresholds (dotted lines) ascertained from 1,000 permutations. Five hundred SNPs against the heritability of 29 traits are plotted. [file 1471-2164-14-897-S3.ppt]
